# Supplementary material for: Mutational spectra are associated with bacterial niche
Source: Nat Commun. 2023 Nov 4;14:7091. doi: 10.1038/s41467-023-42916-w (PMC10625568; doi:10.1038/s41467-023-42916-w)
Supplement: Supplementary file 1 — Supplementary Information [file 41467_2023_42916_MOESM1_ESM.pdf]

Supplementary Note 1 (Dataset sources)

We calculated SBS spectra for 84 phylogenetic clades from 31 diverse bacterial species. In each case, we used whole genome sequences from one or more previous publications. Below, we describe the niches, population structure, sources of genome sequences and the data processing applied to each dataset.

Burkholderia cenocepacia

Causes chronic lung infections in patients with Cystic Fibrosis (CF)[1]. Transmission occurs directly and indirectly between CF patients, although strategies to reduce patient-to-patient transmission were introduced in 2003[1]. While *B. cenocepacia* is diverse, genome sequencing studies have identified clusters of isolates from multiple patients consistent with historical transmission[2]. We obtained all *B. cenocepacia* genome assemblies from GenBank and shredded these to FASTQ files that were mapped against a *B. cenocepacia* reference. Phylogenetic reconstruction showed several clusters of closely related sequences. The majority of sequences in these clusters are from CF patients in Vancouver[2]. The clusters include multiple samples from individual patients and all clusters contain multiple patients with first samples preceding introduction of strategies to reduce transmission. The mutations acquired within each cluster were therefore likely acquired during chronic lung infections and we consider lung to be the major niche for these clusters. We included three clusters in the spectrum calculation and excluded long branches that are likely due to knockout of mismatch repair during chronic infection[3].

Burkholderia pseudomallei

Replicates within the rhizosphere and surface groundwater, from which it can infect animals and humans[4,5]. Person-to-person transmission is rare[5,6]. We therefore consider the environment to be the major replication niche. The overall population is divided into five groups, termed groups 1-5, based on phylogenetic clustering, with the same overall groupings inferred from both chromosomes[5]. We calculated spectra for each of the five groups separately; as chromosome 1 and chromosome 2 spectra were highly similar, chromosome 1 spectra were used for further analyses. We obtained each dataset from previous study authors[5] as an alignment of whole genome sequences produced through mapping to a species reference genome.

Campylobacter jejuni

Colonises the gastrointestinal tract of many animal species[7]. Prevalent cause of gastroenteritis in humans upon exposure to animals or their products, contaminated environments or, rarely, person-to-person transmission[7,8]. We therefore consider *C. jejuni* to be gastrointestinal. We obtained a post-gubbins variable sites alignment from previous study authors[9]; as the dataset was diverse, we ran FastBAPS to identify clusters. Five clusters containing a reasonable number of sequences and diversity were extracted. We calculated a mutational spectrum for each cluster separately.

Cutibacterium acnes

Common commensal found across the surface of dry, moist and sebaceous human skin[10], particularly in sebaceous regions of the face and upper body[11]. We therefore consider skin to be the major niche for *C. acnes*. We obtained all *C. acnes* genome assemblies from GenBank and shredded these to FASTQ files before mapping against a species reference.

Enterococcus faecalis

Generalist organism that is ubiquitous in the gastrointestinal tract of animals and insects[12,13]. We therefore consider *E. faecalis* to primarily inhabit a gut niche in multiple hosts. The *E. faecalis* population was previously divided into 173 PopPUNK clusters[12]. We obtained post-gubbins variable sites alignments from previous study authors[12] for three of these clusters and calculated mutational spectra for each cluster separately.

Escherichia coli

Widespread in the gastrointestinal tract of warm-blooded animals[14]. While *E. coli* is often commensal, variants can cause severe intestinal and extraintestinal disease. This has led to the description of eight pathotypes which exhibit preference for different infection sites[15]. The population has previously been divided into PopPUNK clusters that can be assigned pathotypes based on their isolation sources[15]. We obtained datasets for four PopPUNK clusters belonging to different pathotypes: two gastrointestinal clusters, one extraintestinal cluster found in blood and the urinary tract, and one cluster found across these niches. These isolates were obtained as assemblies that were shredded before mapping against a cluster reference.

Shigella sonnei and *Shigella flexneri* are human-adapted gastrointestinal clades within *E. coli*[16]. We therefore consider *Shigella* clades to be gastrointestinal. The *S. sonnei* population can be divided into several major lineages that diverged within the past several hundred years[16]. We examined three of these lineages which we obtained as FASTQ files and mapped against a species reference. *S. flexneri* has been divided into seven phylogenetic groups[17]. We examined phylogenetic group 3 and obtained data as FASTQ files which were mapped against a species reference.

Haemophilus influenzae

Prevalent as a commensal in the human upper respiratory tract[18,19]. While systemic and lower respiratory tract infections can occur, we consider the upper respiratory tract to be the major niche for *H. influenzae*. The species is classified based on production and antigenicity of the polysaccharide capsule. Non-capsule expressing isolates are defined as nontypeable *H. influenzae* while capsule-producing strains are divided into six serotypes termed a through f[19]. Serotype B *H. influenzae* (Hib) has been highly prevalent. We obtained a dataset of Hib sequences from multiple sources that was previously used to develop and test a serotype prediction tool[19]. These samples were obtained as assemblies that were shredded to FASTQ files prior to mapping against a Hib reference.

Klebsiella pneumoniae

Klebsiella pneumoniae is a commensal and environmental organism that can cause a range of opportunistic infections (predominantly in immunocompromised individuals). Of the four species within the *K. pneumoniae* species complex, *K. pneumoniae* sensu stricto causes the majority of human infections and is divided into over 250 sequence types (STs), with only a small number of STs causing most human cases[20]. We calculated spectra for five STs (11, 15, 101, 147 and 258/512), each of which is highly prevalent, geographically widespread and associated with carbapenem resistance[20]. *K. pneumoniae* commonly colonises mucosal surfaces in the human gastrointestinal tract and nasopharynx[21–24] and we therefore treat these sites as major replication niches for these STs. It is possible that substantial replication also occurs on environmental surfaces and during infections of sites including the urinary tract. We used isolates sequenced across multiple previous studies and obtained these samples as FASTQ files and assemblies. Assemblies were shredded to FASTQ files before all samples were mapped against a ST-specific reference.

Legionella pneumophila

Lives in aquatic habitats within amoeba, ciliated protozoa and other protists[25]. Can infect human lungs upon inhalation. However, as human-to-human transmission at most occurs very rarely[26], we consider aquatic protists to be the major niche for *L. pneumophila*. The population is currently divided into over 2000 sequence types (STs) with large variation in prevalence[26]. We calculated spectra of ST1 (one of the most prevalent STs in Europe and North America) and a group of low prevalence STs (ST51, ST87, ST376, ST454, ST578 and ST611)[26]. Isolates were obtained as FASTQ files that were mapped against a local reference.

Mycobacterium abscessus

We used *M. abscessus* SBS spectra calculated previously[27]. In this previous study, we identified seven dominant circulating clones (DCCs) amongst the *M. abscessus* phylogenetic diversity. These DCCs are commonly isolated from lung infections of individuals with and without Cystic Fibrosis and exhibit features consistent with person-to-person transmission, including clustering of geographically diverse isolates[27,28].

However, the major replication niche(s) of the DCCs remains controversial. The remainder of the *M. abscessus* species tree consists of diverse isolates consistent with sampling from a diverse environmental reservoir; we consider these isolates to be environmental and refer to these isolates as environmental *M. abscessus*[27,28]. We used SBS spectra that we previously calculated for the DCCs and environmental *M. abscessus*[27].

Mycobacterium avium
A primarily environmental bacterium that can be isolated from soil and water sources[29]. We therefore consider *M. avium* to be environmental. The population is divided into three major clusters that are further divided into subclusters[30]. We calculated the spectrum of the *M. avium* species using a dataset containing samples from all three clusters[30]. Isolates were obtained as genome assemblies and shredded to FASTQ files prior to mapping.

Mycobacterium canettii
M. canettii is believed to be an environmental bacterium although most isolates are from human infections[31,32]. Lineages are not currently defined amongst the limited number of sequenced *M. canettii* genomes, although a recently emerged outbreak clade has been identified[31]. We calculated the mutational spectrum of the available diversity in *M. canettii*. Samples were obtained as FASTQ files that were mapped against a complete genome from the closely related *Mycobacterium tuberculosis*.

Mycobacterium chimaera
An environmental bacterium that can be isolated from multiple environmental sources[33]. Can cause opportunistic pulmonary infections and caused a large outbreak of invasive infections associated with heater cooler units (HCUs)[34,35]. We therefore consider *M. chimaera* to be environmental. Isolates associated with the HCU outbreak form a single clade[35]. We calculated the *M. chimaera* mutational spectrum using all available genome sequences. Samples were obtained as FASTQ files that were mapped against a species reference.

Mycobacterium intracellulare
Environmental bacterium that can be isolated from soil and water sources[34]. We therefore consider *M. intracellulare* to be environmental. The population structure of *M. intracellulare* has not been well defined. We calculated the mutational spectrum of the *M. intracellulare* species using all available genome sequences. Samples were obtained as FASTQ files that were mapped against a species reference.

Mycobacterium kansasii
Environmental bacterium that can be isolated from multiple environmental sources[34], although water is likely a major reservoir for *M. kansasii* infections[36]. A previous population genomics study showed that most isolates cluster within a single clade termed the *M. kansasii* main cluster (MKMC)[36]. The remaining isolates fall on long terminal phylogenetic branches and are not closely related to other isolates, as expected of diverse environmental samples. We therefore consider the non-MKMC region of the *M. kansasii* tree to be environmental. The MKMC exhibits characteristics potentially consistent with patient-to-patient transmission, including closely related isolates from distant geographical locations, but the role of patient-to-patient transmission compared with environmental acquisition is currently unclear[36]. We therefore consider the major niche of the MKMC to be unclear. We calculated mutational spectra for the non-MKMC and MKMC regions of the tree separately. Samples were obtained as assemblies that were shredded to FASTQ files prior to mapping against a species reference.

Mycobacterium leprae
M. leprae is known to infect the respiratory system, skin and peripheral nervous system of humans[37]. Transmission occurs between people through droplets from the nose and mouth (https://www.who.int/news-room/fact-sheets/detail/leprosy, last accessed 20th April 2023). We therefore consider the major niche of *M. leprae* to be the human respiratory system. The *M. leprae* population is

currently divided into four SNP types and 16 SNP subtypes[38]. We calculated the mutational spectrum of the *M. leprae* species. Samples were obtained as FASTQ files that were mapped against a species reference. The dataset includes several known hypermutator strains with mutations in the *nth* gene[38]; mutations on the tip phylogenetic branches representing hypermutator strains were excluded from the spectrum.

Mycobacterium tuberculosis
The *M. tuberculosis* complex (MTBC) consists of human and animal pathogens. Human infections are currently divided into lineages 1 through 9 which show highly different prevalence and phylogeographical patterns[39]. The human lineages predominantly infect the lungs and transmit directly from one person to another through inhalation of infectious particles. The animal lineages analysed here form a closely related monophyletic clade and are each associated with infection of specific host species: *M. bovis* in cattle, *M. caprae* in sheep and goats and *M. orygis* in antelope[39]. The lung is likely a major target of these infections[40,41]. We therefore consider the lung to be the major niche for *M. tuberculosis* lineages. We calculated mutational spectra for the major human lineages L1-L7 (L8 and L9 were not analysed due to a limited number of genomes) and for the animal lineages *M. bovis, M. caprae* and *M. orygis*. Samples were obtained as FASTQ files that were mapped against a local reference.

Neisseria gonorrhoea
Predominantly colonises the genital mucosa[42]. We therefore consider *N. gonorrhoea* to be genital. Multiple clades have previously been identified[43]. We calculated the mutational spectrum of the *N. gonorrhoea* species using available whole genome sequences. These were obtained as FASTQ files that were mapped against a species reference.

Neisseria meningitidis
Asymptomatically colonises the oropharynx in roughly 10% of humans and transmits from one person to another through inhalation of respiratory secretions and saliva[44]. We analyse two respiratory clusters of *N. meningitidis* and an additional cluster associated with urethritis. The urethritis cluster likely predominantly resides within the upper respiratory tract but can infect the urogenital tract upon exposure[45]. We therefore consider the upper respiratory tract to be the major niche for the two respiratory *N. meningitidis* clusters and the upper respiratory and urogenital tracts to be major niches for the urethritis cluster. We calculated the mutational spectra of these three clusters separately. Samples were obtained as FASTQ files and mapped against a local reference.

Pseudomonas aeruginosa
Causes acute and chronic infections in a variety of human tissues, including respiratory infections (particularly in individuals with Cystic Fibrosis (CF)), urinary tract infections, skin and soft tissue infections and keratitis[46]. Can also be identified in a variety of environmental niches, often associated with human activity[47]. The interaction between these different niches and the sources of *P. aeruginosa* infections are not well understood. Many of the genomes we include here were collected from lung infections of individuals with CF. We therefore consider *P. aeruginosa* to have multiple niches including the human lung. *P. aeruginosa* is divided into a large number of sequence types. We calculated mutational spectra for five common sequence types: ST17, ST111, ST146, ST175 and ST253. Samples were obtained as FASTQ files that were mapped against a species reference.

Salmonella enterica serovar Typhimurium
Serovar of non-typhoidal *Salmonella* that causes gastroenteritis, some lineages of which are also a frequent cause of invasive disease in sub-Saharan Africa[48]. We therefore consider Typhimurium to be both gastrointestinal and invasive. Typhimurium is divided into many sequence types[49]. We calculated the Typhimurium mutational spectrum using a dataset containing gastrointestinal and invasive isolates belonging to multiple sequence types[48]. Samples were obtained as a mixture of FASTQ files and assemblies that were initially shredded to FASTQ files. All samples were mapped against a serovar reference.

Staphylococcus aureus
Carried on the skin and in the nasopharynx (upper respiratory tract) in humans. Longitudinal studies have shown that approximately 50-80% of the human population are intermittent *S. aureus* nasal carriers and 12-30% are persistent carriers[50,51]. *S. aureus* has also been detected in a taxonomically and ecologically diverse range of animal hosts. We therefore consider the upper respiratory tract and skin to be the major niches for *S. aureus*. *S. aureus* population structure is highly clonal, with over 90% of known S. aureus genomes being categorized into just four predominant clonal complexes (CC5, CC8, CC398 and CC30)[52]. We calculated mutational spectra for four recently emerged clades of *S. aureus* with different host ranges: ST22 (human)[53], ST239 (human)[54], ST772[55] and CC398 (livestock and human)[56]. Samples were obtained as FASTQ files that were mapped against a local reference.

Staphylococcus epidermidis
Carried on the skin of virtually all humans[57], predominantly within moist skin sites[10]. We therefore consider skin (and in particular moist skin sites) to be the major niche for *S. epidermidis*. Multiple levels of phylogenetic classification exist for *S. epidermidis*: over 80 STs have been described, most of which only contain a small number of sequenced genomes; the STs are grouped into CCs; the CCs are further grouped into phylogenetic groups of which groups A, B and C are most prevalent[57,58]. We analysed phylogenetic groups A, B and C. These samples were obtained as assemblies that were shredded into FASTQ files prior to mapping against a species reference.

Staphylococcus haemolyticus
Widespread skin commensal found in the axilla, perineum and inguinal areas that can also cause septicaemia, peritonitis, otitis and urinary tract infections[59,60]. We therefore consider localised regions of the skin to be the major niche for *S. haemolyticus*. Multiple clades have been defined within *S. haemolyticus* based on genomic variation[61]. We calculated the mutational spectrum of the species using a dataset of geographically diverse isolates[61]. Samples were obtained as FASTQ files that were mapped against a species reference.

Streptococcus agalactiae
Commensal bacterium that colonizes the human gastrointestinal and genital tracts in a high proportion of adults[62,63]. Can cause invasive disease through septicaemia and meningitis in young children[64]. We consider the human gastrointestinal and genital tracts to be the major replication niches for *S. agalactiae*. *S. agalactiae* isolates cluster into clonal complexes (CCs). We analysed the five CCs associated with the majority of carriage and clinical isolates: CC1, CC10, CC17, CC19 and CC23[64,65]. These samples were obtained as whole genome alignments from previous study authors[64].

Streptococcus equi
Infects the equine upper respiratory tract and transmits from one individual to another upon bacterial shedding via the nasopharynx[66,67]. We therefore consider the equine upper respiratory tract to be the major niche for *S. equi*. The global population of *S. equi* has been divided into BAPS clusters and sequence types[67]. We calculated the mutational spectrum of the complete *S. equi* clade using a geographically diverse dataset[67]. Samples were obtained as FASTQ files that were mapped against a clade reference.

Streptococcus pneumoniae
Colonizes the mucosal surfaces of the human upper respiratory tract[68]. Carried in the upper respiratory tract by a high proportion of children and adults and transmits from one person to another[68]. We therefore consider the human upper respiratory tract to be the dominant niche for *S. pneumoniae* lineages. Large scale genome sequencing has classified the *S. pneumoniae* population into over 600 Global Pneumococcal Sequence Clusters (GPSCs) which vary greatly in prevalence[69]. We analysed 5 prevalent GPSCs (GPSC1, GPSC2, GPSC3, GPSC6 and GPSC54) which were obtained from previous study authors[69] as recombination-stripped variable sites alignments.

Streptococcus pyogenes

Common cause of human upper respiratory tract and skin infections[70]. The mucosal epithelium and oropharynx are the primary replication sites of *S. pyogenes* and transmission occurs directly from one person to another through respiratory droplets or skin contact[70]. We therefore consider the upper respiratory tract and skin to be the major niches for *S. pyogenes*. Analysis of the core and accessory genome has divided *S. pyogenes* into roughly 300 phylogroups[71]. We analysed the four most prevalent phylogroups[71]. Samples were obtained as either FASTQ files or assemblies (which we shredded to FASTQ files) and were mapped against a phylogroup-specific reference.

Yersinia pestis

Predominantly exists in sylvatic infection cycles between many species of rodents and their fleas[72,73]. Additional transmission pathways also occur to predatory birds and mammals and to humans, where person-to-person transmission is possible[72]. The bacterium replicates within the flea gastrointestinal tract[74]. We therefore consider rodents and fleas, including the gastrointestinal tract and blood, to be the major niches for *Y. pestis*. The *Y. pestis* population is divided into several branches on the basis of mutations[75]. We calculated the mutational spectrum of the species using a geographically diverse dataset that captures the currently known sequence diversity[75]. Samples were obtained as assemblies that were shredded to FASTQ files before mapping against a species reference.

Supplementary Note 2 (Examination of the *Mycobacterium kansasii* MKMC clade)

The genetic diversity within the *M. kansasii* species can be divided into the *M. kansasii* main cluster (MKMC) and non-MKMC isolates[36]. The MKMC emerged in the early 1900s and contains closely related isolates that have been collected from 11 countries on six continents, while the non-MKMC isolates are highly diverse[36]. Previous analyses have suggested that *M. kansasii* infections are acquired independently from environmental water sources[36]. We found that the non-MKMC isolates exhibit a mutational spectrum consistent with the environmental niche (**Fig. 4, Fig. S18**). However, the MKMC spectrum exhibits characteristics of both environmental and lung spectra (**Fig. 4, Fig. S18**). The spectra results therefore suggest that the MKMC lives within and can be acquired from both the environment and the lung. We investigated this possibility further through transmission network analyses and gene burden testing.

We identified probable and possible transmission events within the MKMC using SNP distance cutoffs previously established for *Mycobacterium abscessus*[28,76] (which exhibits a highly similar substitution rate to the MKMC[27,36]). Reconstructing transmission networks at these SNP distances showed that almost all transmission linkages are within the MKMC (**Fig. S19**). We observe large numbers of transmission linkages at each cutoff, including many international linkages which cannot be explained by shared water sources (**Fig. S19**).

Gene burden testing identified 24 genes containing more missense and nonsense mutations than expected given their length and the number of observed mutations (**Fig. S20**). These genes include two adjacent *tetR* family members (*tetR1* and *tetR2*) that were previously observed to be under positive selection in the MKMC[36]. We identified 14 independent mutations of *tetR1*/*tetR2* within the MKMC, six of which occurred on internal phylogenetic branches leading to multiple isolates (**Fig. S21**). 26% of MKMC isolates contain a missense or nonsense mutation in *tetR1* or *tetR2* and mutations in these genes occur more often on internal phylogenetic branches than expected ($p = 0.017$, Fisher exact test).

Genes within the *tetR* family are involved in adaptation of *M. abscessus* to the human lung[77]. We therefore hypothesised that *tetR* mutations on internal phylogenetic branches occurred within the human lung and transmitted onwards to multiple individuals through human-to-human lung transmission. Supporting this, we observe multiple clades with *tetR1* or *tetR2* mutation that were collected on multiple continents (**Fig. S21**), including a clade we termed "MKMC tetR1/2 clade 1" that was sampled in China, Japan and Australia. We were able to investigate the mutational spectrum of the internal phylogenetic branches of this clade and

found high levels of C>A mutations and low levels of T>C mutations (**Fig. S21B**), strongly supporting the internal phylogenetic branches of MKMC tetR1/2 clade 1 being within the lung.

Together, these data strongly support a significant proportion of MKMC infections being acquired through human-to-human lung transmission, further supporting the patterns observed in the mutational spectra.

Supplementary Figures

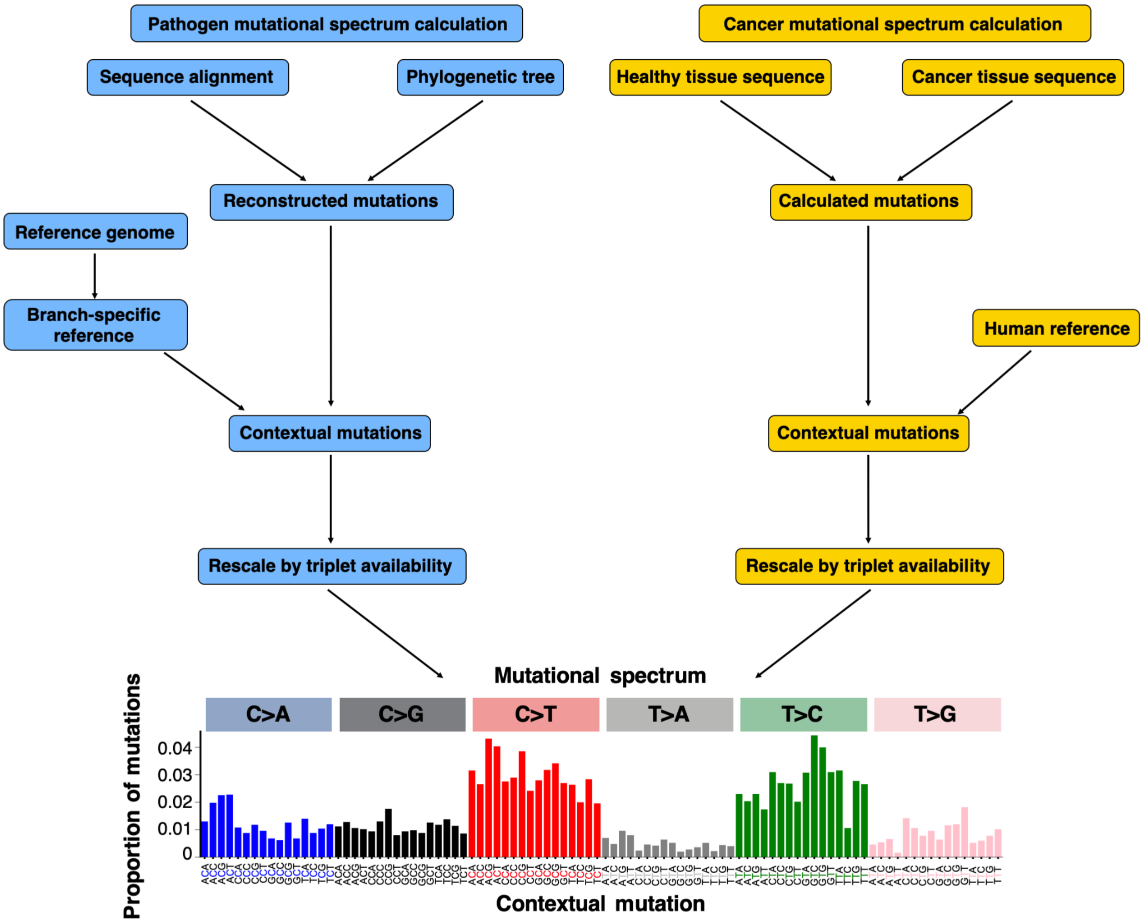

Figure S1. Summary of the pipeline used to calculate pathogen SBS spectra and comparison to cancer SBS spectrum calculation. To calculate pathogen SBS spectra, we initially reconstruct directional mutations from an alignment of genetic sequences and a phylogenetic tree. The context of each mutation is identified from a reference genome that is updated at each phylogenetic node to incorporate mutations that have occurred preceding the node within the phylogenetic tree, thereby enabling identification of the context of each mutation within the branch on which it occurred. Contextual mutations are rescaled by triplet availability within the reference genome to enable comparison between bacteria. We have implemented pathogen mutational spectrum calculation in the open-source software tool MutTui (https://github.com/chrisruis/MutTui).

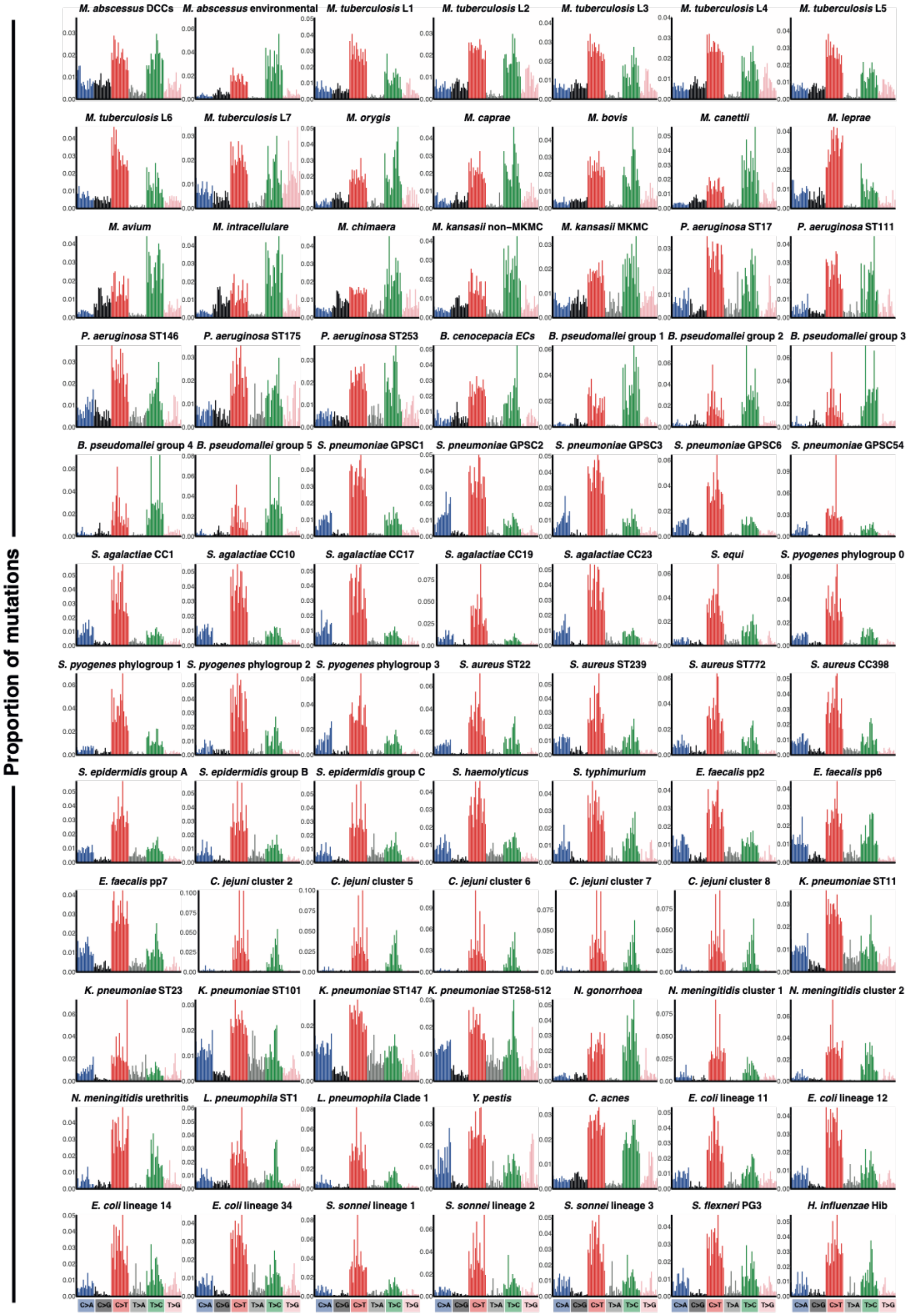

Figure S2. SBS spectra reconstructed from 84 phylogenetic groups across 31 bacterial species. We assembled datasets containing whole genome sequence alignments and phylogenetic trees from previous publications and reconstructed SBS spectra as described in **Fig. S1**. Source data are provided as a Source Data file.

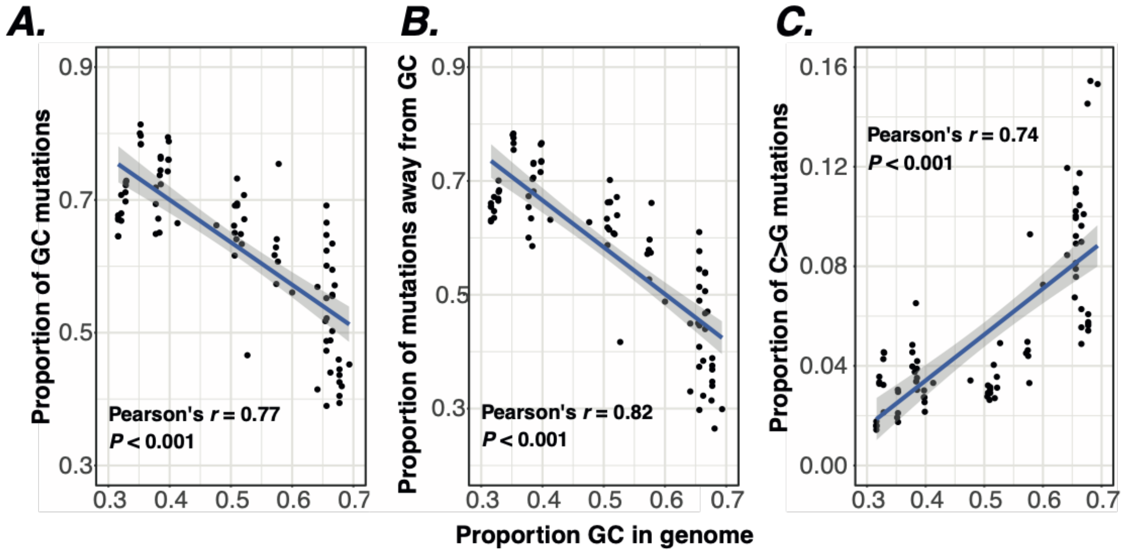

Figure S3. Correlation between genomic G+C content and GC base mutations. Correlation between the proportion of G and C nucleotides in the genome with (**A**) the proportion of mutations of GC base pairs (including C>A, C>G and C>T) (P < 2.2E-16), (**B**) the proportion of mutations from a GC base pair to an AT or TA base pair (including C>A and C>T) (P < 2.2E-16) and (**C**) the proportion of C>G mutations (P = 3.26E-16). n = 84 SBS spectra in each case; all SBS spectra in **Fig. S2** are included. Correlation p-values were calculated using Pearson's product moment correlation coefficient. Source data are provided as a Source Data file.

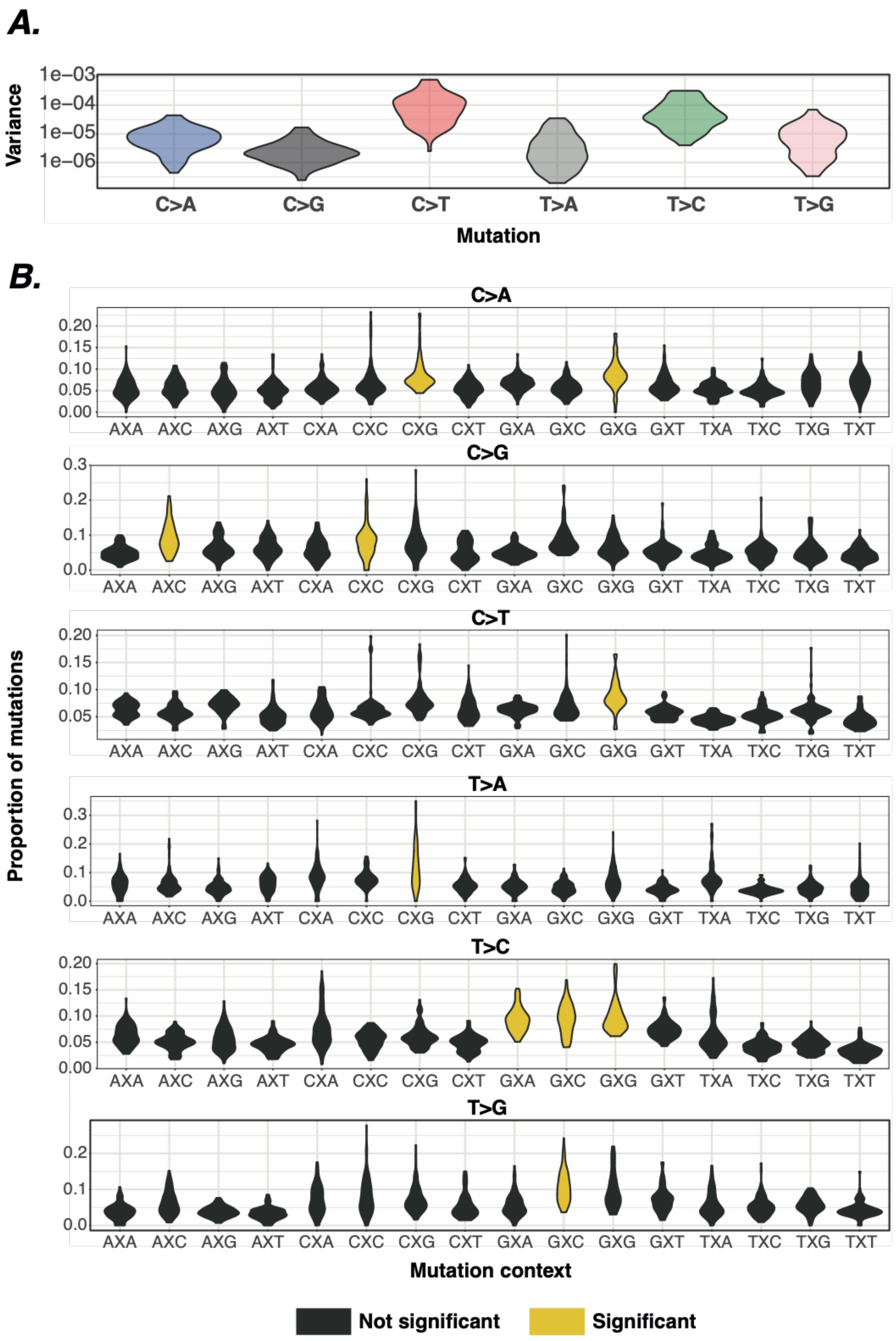

Figure S4. Summaries of mutation contexts across datasets. (**A**) Distribution of variance of the 16 contextual mutation proportions within each mutation type across the 84 SBS spectra. C>T and T>C exhibit elevated context-specificity (Tukey HSD corrected two-way ANOVA *P* < 0.001). (**B**) Distribution of contextual mutation proportions within each mutation type across the 84 SBS spectra. Significant contexts exhibit median context proportion at least 2.5 median average deviations away from the median proportion across all contexts within the mutation type. n = 84 SBS spectra in each case. Source data are provided as a Source Data file.

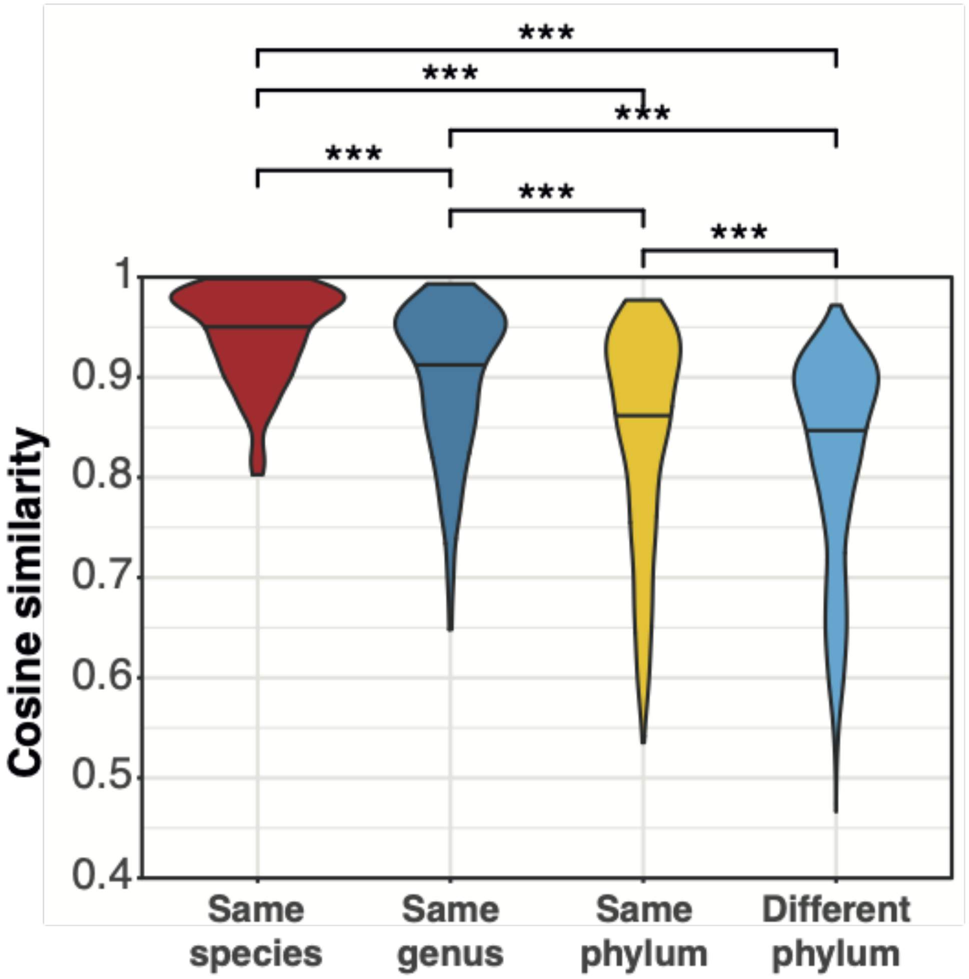

Figure S5. Correlation between phylogenetic relatedness and SBS spectrum similarity. We calculated the cosine similarity between all SBS spectrum pairs and split based on taxonomic relationship. Cosine similarities are only placed in the lowest taxonomic match, for example if spectra are from the same species, they will not be included in the same genus comparisons. Significance was calculated using Tukey HSD corrected two-way ANOVA between the distributions, *** - $P < 0.001$ (p-values: same species vs same genus = 7.45E-5; same species vs same phylum < 2.2E-16; same species vs different phylum < 2.2E-16; same genus vs same phylum < 2.2E-16; same genus vs different phylum < 2.2E-16; same phylum vs different phylum = 3.0E-7). n = number of comparisons (sample species = 157; same genus = 230; same phylum = 831; different phylum = 2268). Source data are provided as a Source Data file.

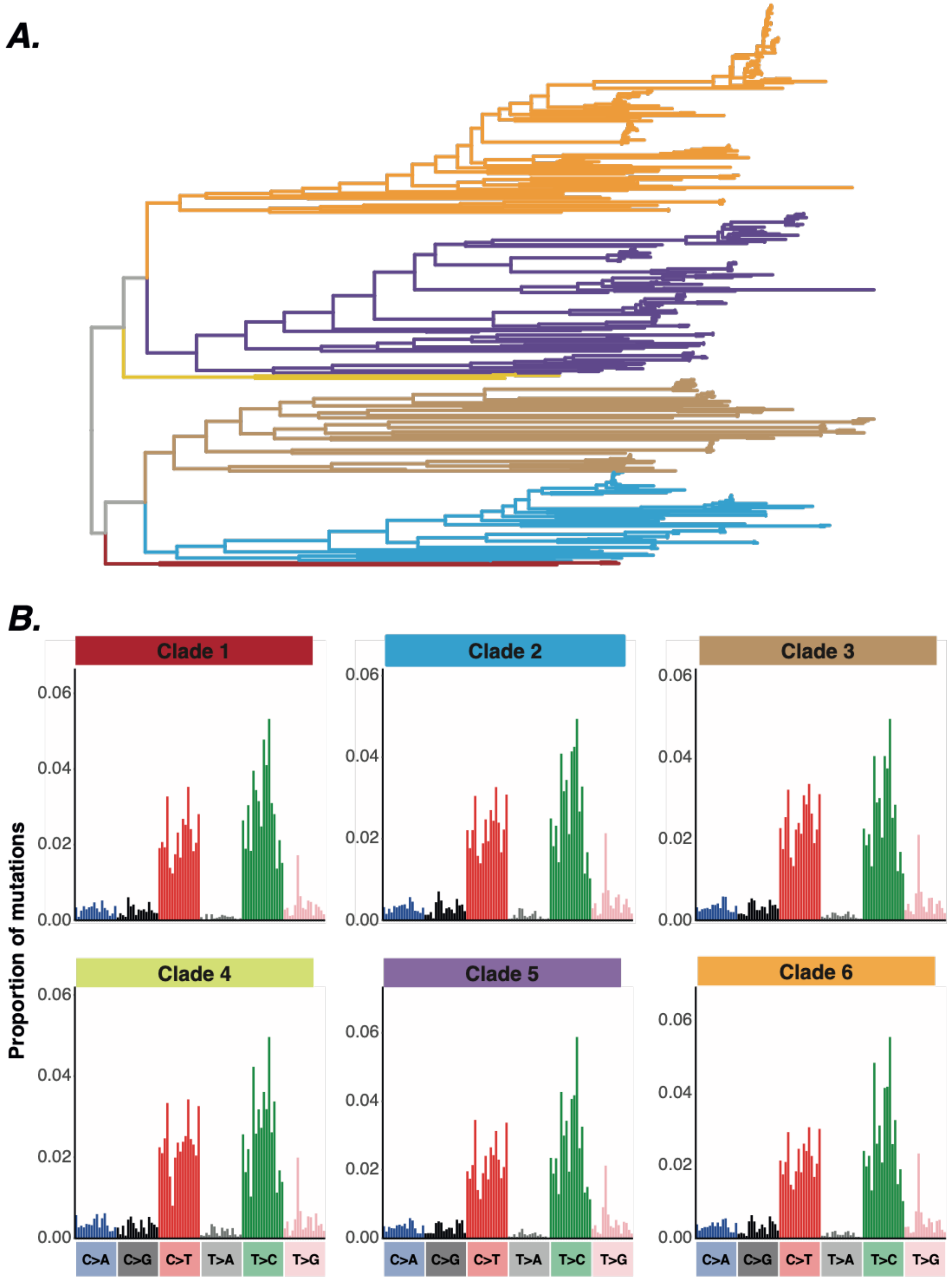

Figure S6. Comparison of SBS spectra between *Neisseria gonorrhoeae* clades. (**A**) Phylogenetic tree of 412 *N. gonorrhoeae* isolates used to reconstruct the SBS spectrum. The tree is coloured by clade based on phylogenetic clustering. The *N. gonorrhoeae* SBS spectrum included in other analyses was calculated across the complete tree. (**B**) SBS spectrum of each of the six clades highlighted in panel **A**. SBS spectra are highly similar between clades, cosine similarity >0.98 between all pairs. Source data are provided as a Source Data file.

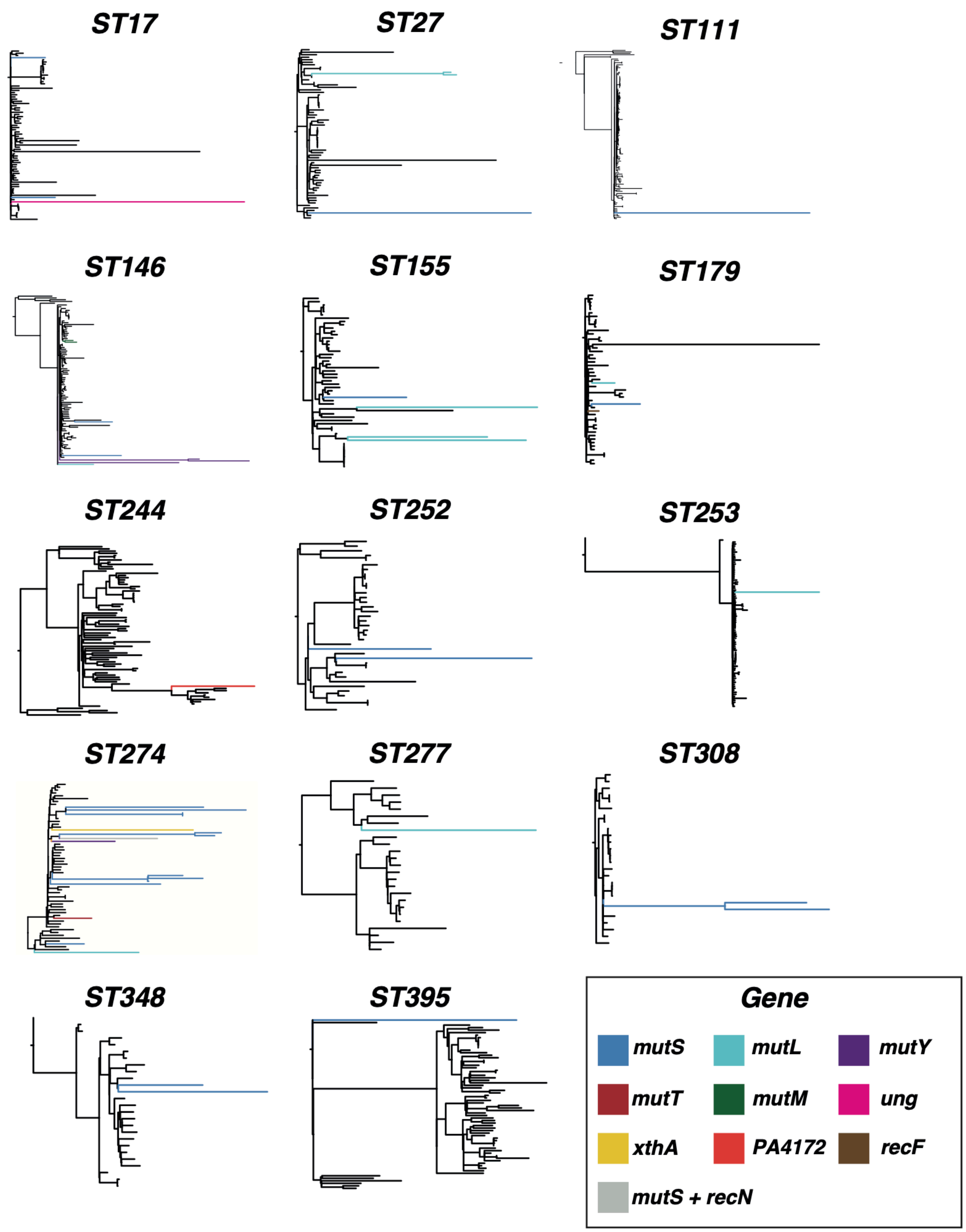

Figure S7. Hypermutator branches in *P. aeruginosa* sequence types. *P. aeruginosa* sequence type phylogenetic trees showing hypermutator branches and the inferred responsible genes. Hypermutator branches were identified based on branch length and the ratio of transition and transversion mutations and are coloured based on the inferred responsible gene. Responsible genes were identified as DNA repair genes exhibiting a mutation on the long phylogenetic branch or ancestral branch. Black branches did not contribute to hypermutator spectra. Sequence types are labelled above the corresponding tree.

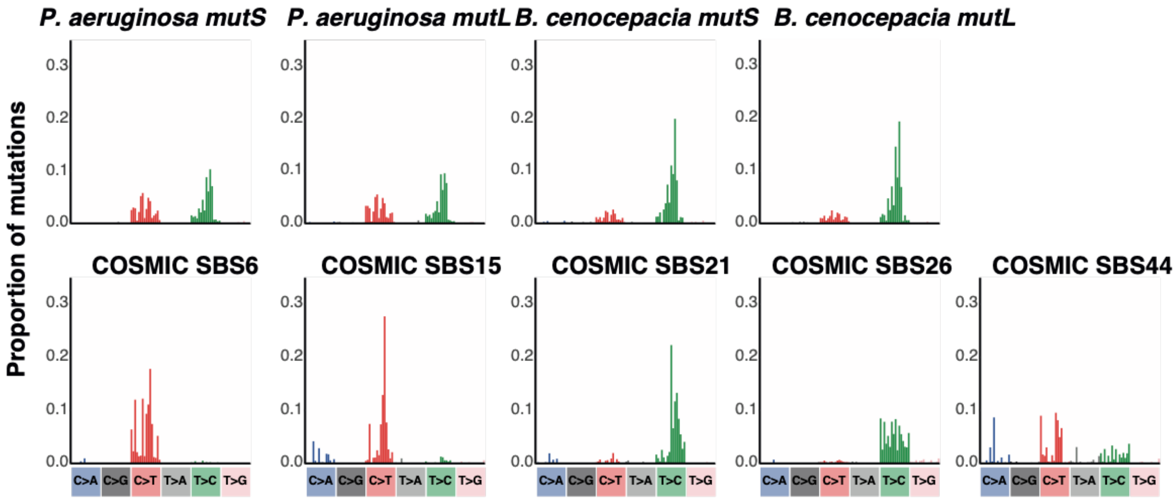

Figure S8. Comparison of MMR signatures between bacteria and humans. The top row shows the MMR signatures extracted from bacterial hypermutator lineages, as in **Fig. 2B**. The bottom row shows the five COSMIC signatures currently associated specifically with defective MMR in humans. Source data are provided as a Source Data file.

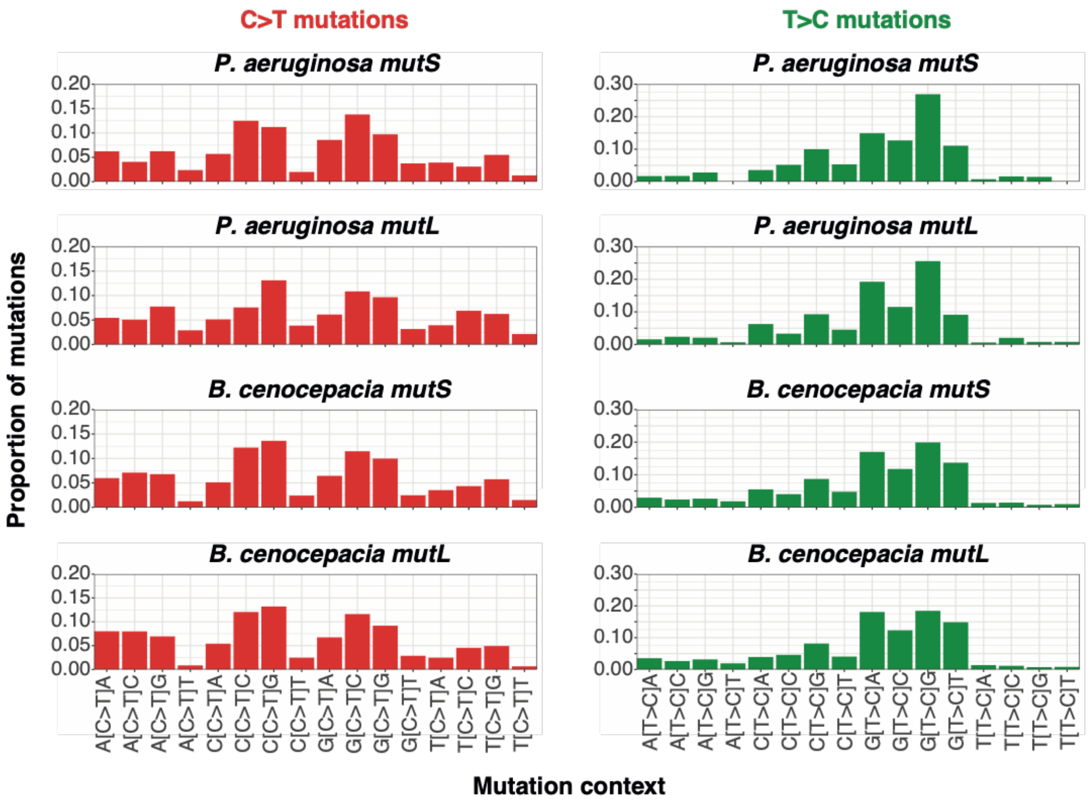

Figure S9. Comparison of contextual C>T and T>C mutations between mismatch repair hypermutators in *P. aeruginosa* and *B. cenocepacia*. The proportion of each contextual mutation within C>T or T>C is shown. Cosine similarity between contextual mutation proportions within C>T or within T>C >0.95 between all pairs. Source data are provided as a Source Data file.

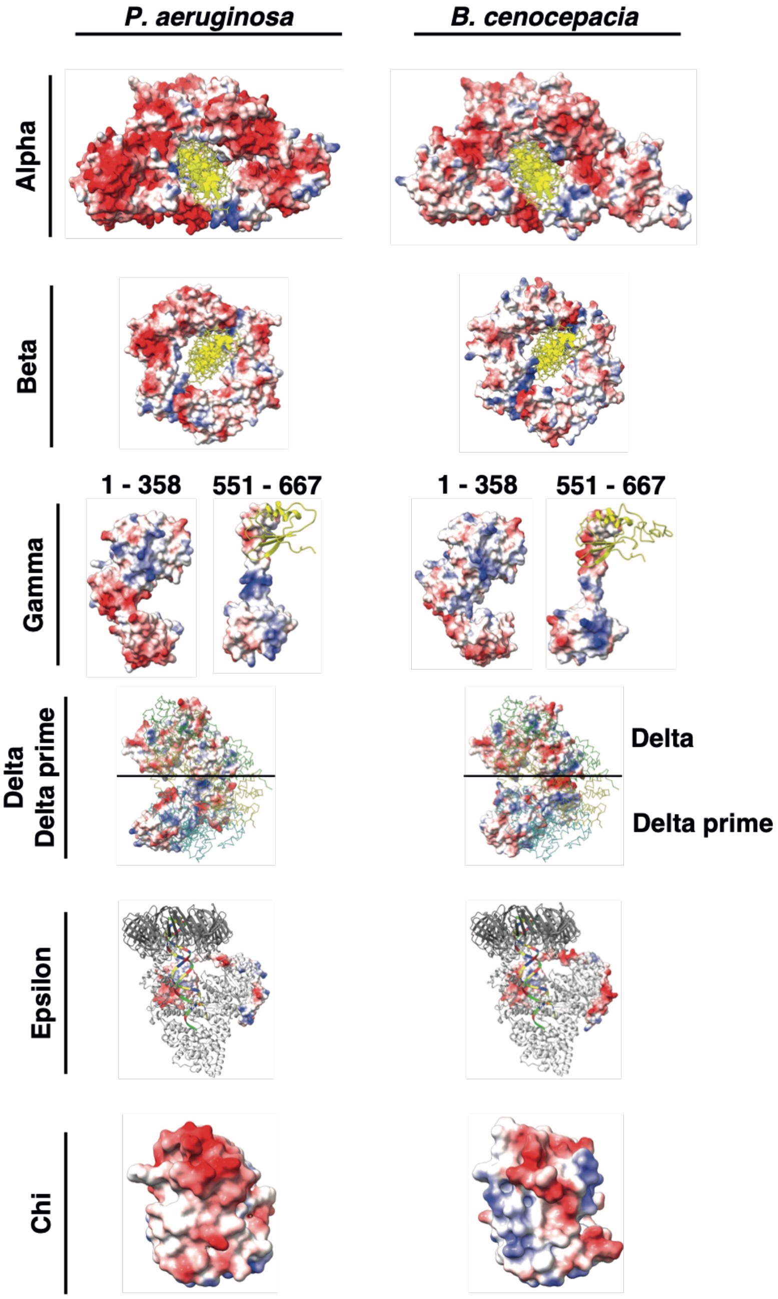

Figure S10. Structural modelling of DNA polymerase III. Predicted models of each subunit of DNA polymerase III in *P. aeruginosa* and *B. cenocepacia* are shown as electrostatic surfaces with negative charges shown in red and positive charges in blue. Alpha and beta subunit predicted models are superposed on the crystal structure of *E. coli* replicative DNA polymerase complex (PDB ID 5FKV) that contains bound DNA (shown in yellow as stick representation). Residues with predicted local distance difference test (pLDDT) score greater than 70 are shown for the gamma subunit. Numbers above the structures indicate included residues. Gamma subunit models include the c-terminal residues of the alpha subunit (yellow ribbon) that interact with the gamma subunit, inferred by superposing the respective subunits onto the cryo EM structure of the *E. coli* replicative DNA polymerase complex (PDB ID 5FKV). Horizontal lines in the delta and delta prime models separate the interface between delta (above the line) and delta prime (below the line) subunits; the interaction interface is inferred by superposing the homology models onto the respective subunits of the crystal structure of the *E.coli* clamp loader complex (PDB ID 1XXI; gamma subunit chains B, C and D shown as c-alpha trace in green, yellow and cyan respectively). Predicted epsilon subunit models are superposed onto the cryo EM structure of the *E. coli* replicative DNA polymerase complex (PDB ID 5FKW; alpha and beta subunits shown as ribbon in light grey and dark grey, respectively, bound DNA shown as coloured ribbon). Overall DNA polymerase III subunit structures are similar between *P. aeruginosa* and *B. cenocepacia* but electrostatic surfaces are distinct.

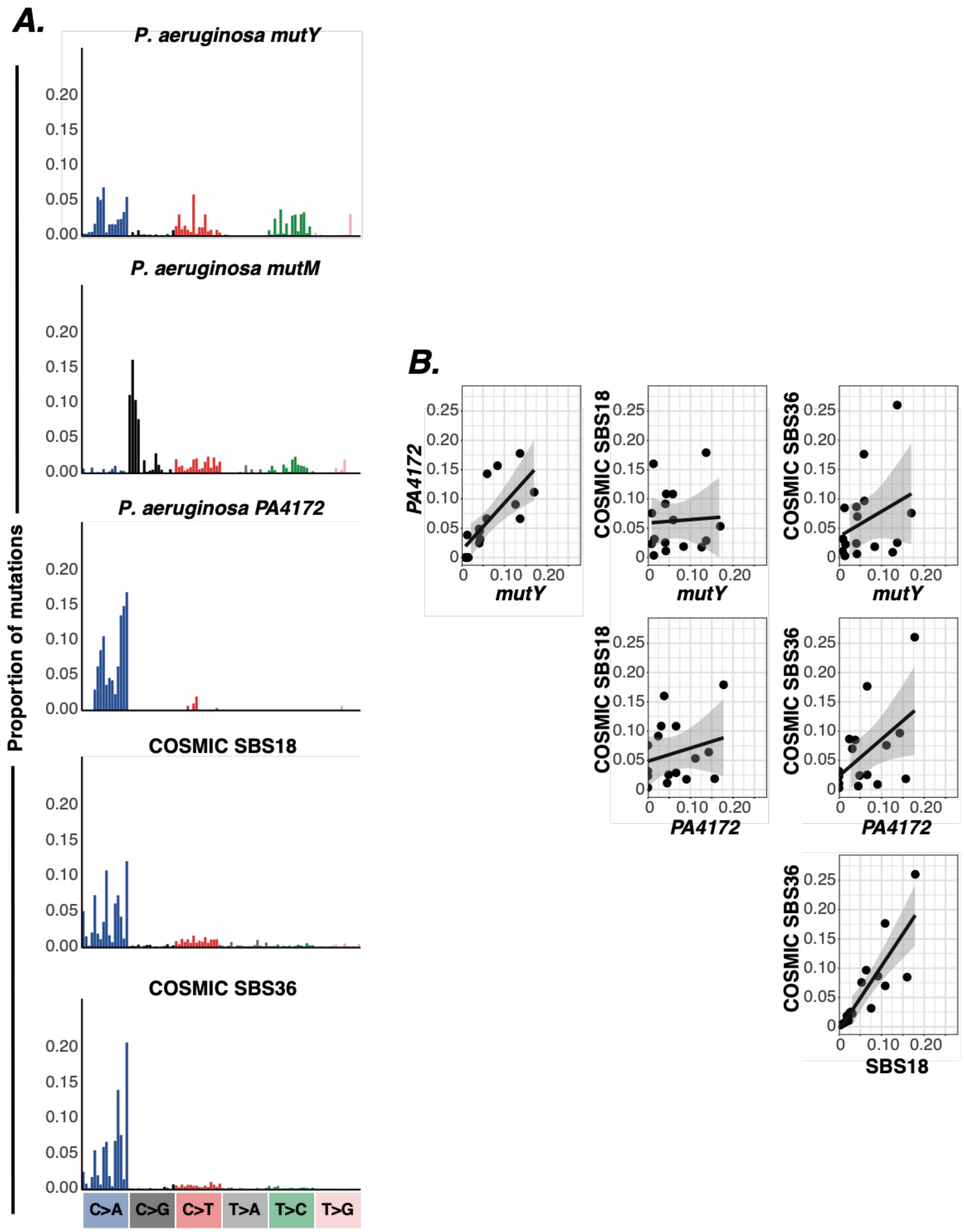

Figure S11. Comparison of GO signatures between bacteria and humans. (A) Mutational signatures of GO genes extracted from bacteria and COSMIC signatures associated with GO genes in humans. (**B**) Regression of the proportion of the 16 contextual mutations within C>A between gene signatures that exhibit C>A mutations. The correlation is significant between *P. aeruginosa mutY* and *P. aeruginosa PA4172* (Pearson's $r = 0.73$; 95% CI: 0.37, 0.9; Benjamini-Hochberg corrected $P = 0.005$) and between COSMIC SBS18 and COSMIC SBS36 (Pearson's $r = 0.84$; 95% CI: 0.59, 0.94; Benjamini-Hochberg corrected $P < 0.001$). Correlation p-values were calculated using Pearson's product moment correlation coefficient. Source data are provided as a Source Data file.

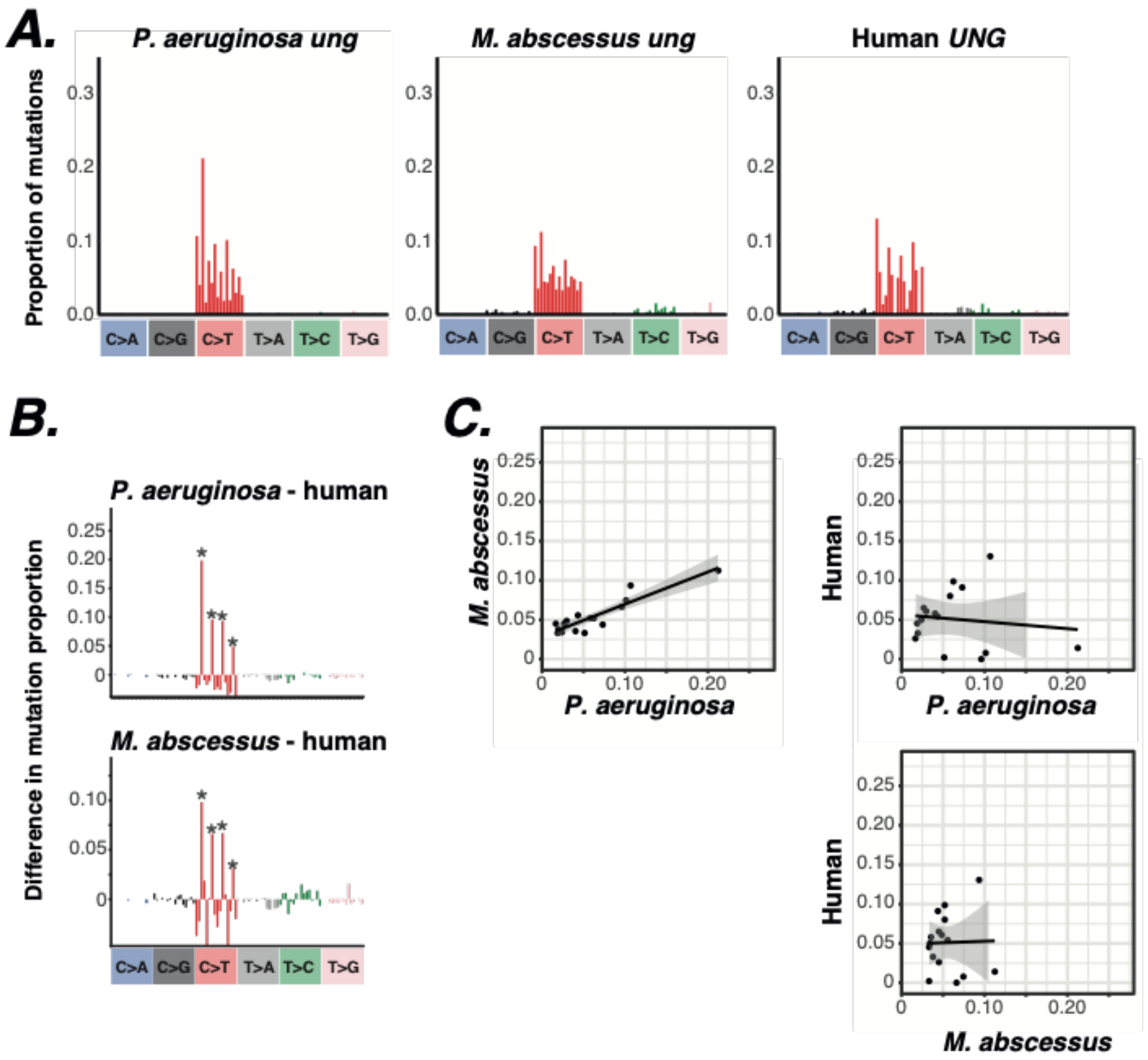

Figure S12. Comparison of *ung* signatures between bacteria and humans. (A) Mutational signatures of *ung* gene hypermutator lineages extracted from bacteria and *in vitro UNG* knockout in human cells. (**B**) Subtraction of the human *UNG* knockout signature from the bacterial *ung* signatures. Asterisks indicate CpG contexts. (**C**) Regression of the proportion of the 16 contextual mutations within C>T between gene signatures. The correlation is significant between *P. aeruginosa ung* and *M. abscessus ung* (Pearson's r = 0.91; 95% CI: 0.75, 0.97; Benjamini-Hochberg corrected $P < 0.001$). Correlation p-values were calculated using Pearson's product moment correlation coefficient. Source data are provided as a Source Data file.

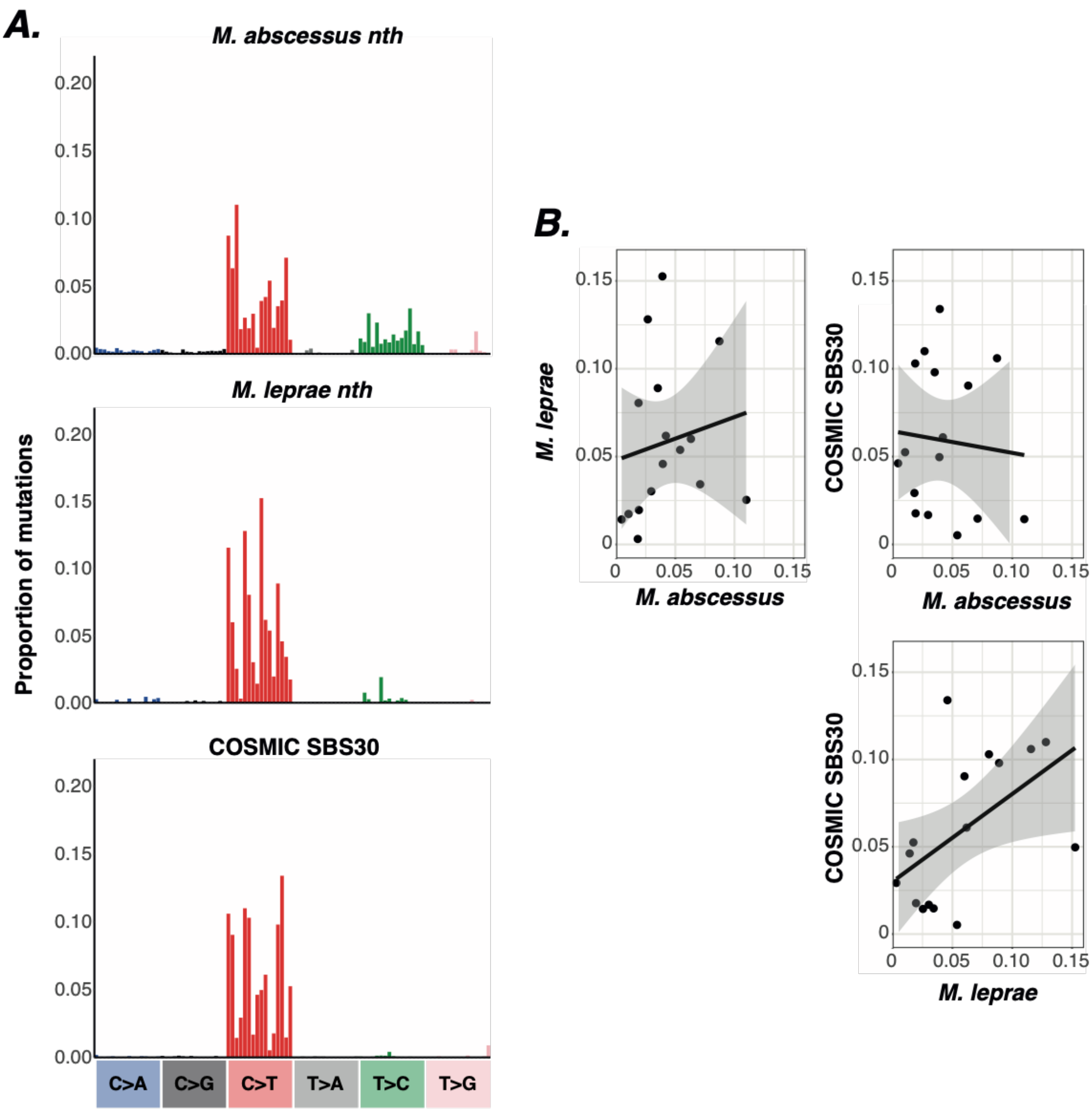

Figure S13. Comparison of *nth* signatures between bacteria and humans. (A) Mutational signatures of *nth* gene hypermutator lineages extracted from bacteria and COSMIC SBS30 which is associated with *in vitro* knockout of the *nth* homologue *NTHL1* in human cells. (**B**) Regression of the proportions of the 16 contextual mutations within C>T between gene signatures. None of the correlations are statistically significant. Correlation p-values were calculated using Pearson's product moment correlation coefficient. Source data are provided as a Source Data file.

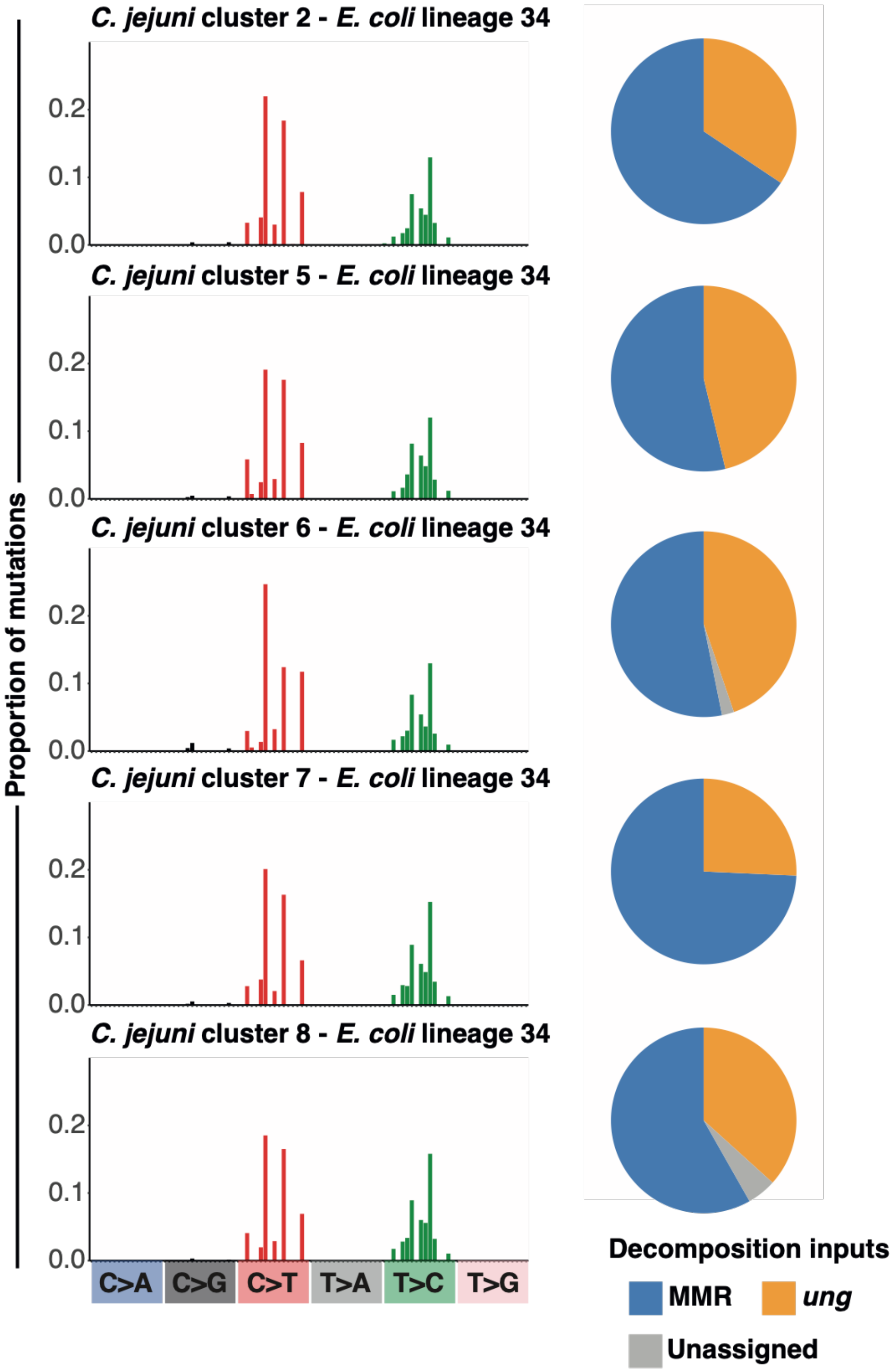

Figure S14. Signatures of DNA repair deficiency in *C. jejuni*. Spectrum plots show the SBS mutations elevated in the respective *C. jejuni* cluster compared with *E. coli* lineage 34 which occupies a similar niche. Due to the shared niche, these elevated mutations are likely the result of processes ongoing within *C. jejuni*. Pie charts show the proportion of elevated mutations assigned to the respective bacterial DNA repair signature in a decomposition analysis into the full set of extracted bacterial DNA repair signatures in **Fig. 2B-D**. Source data are provided as a Source Data file.

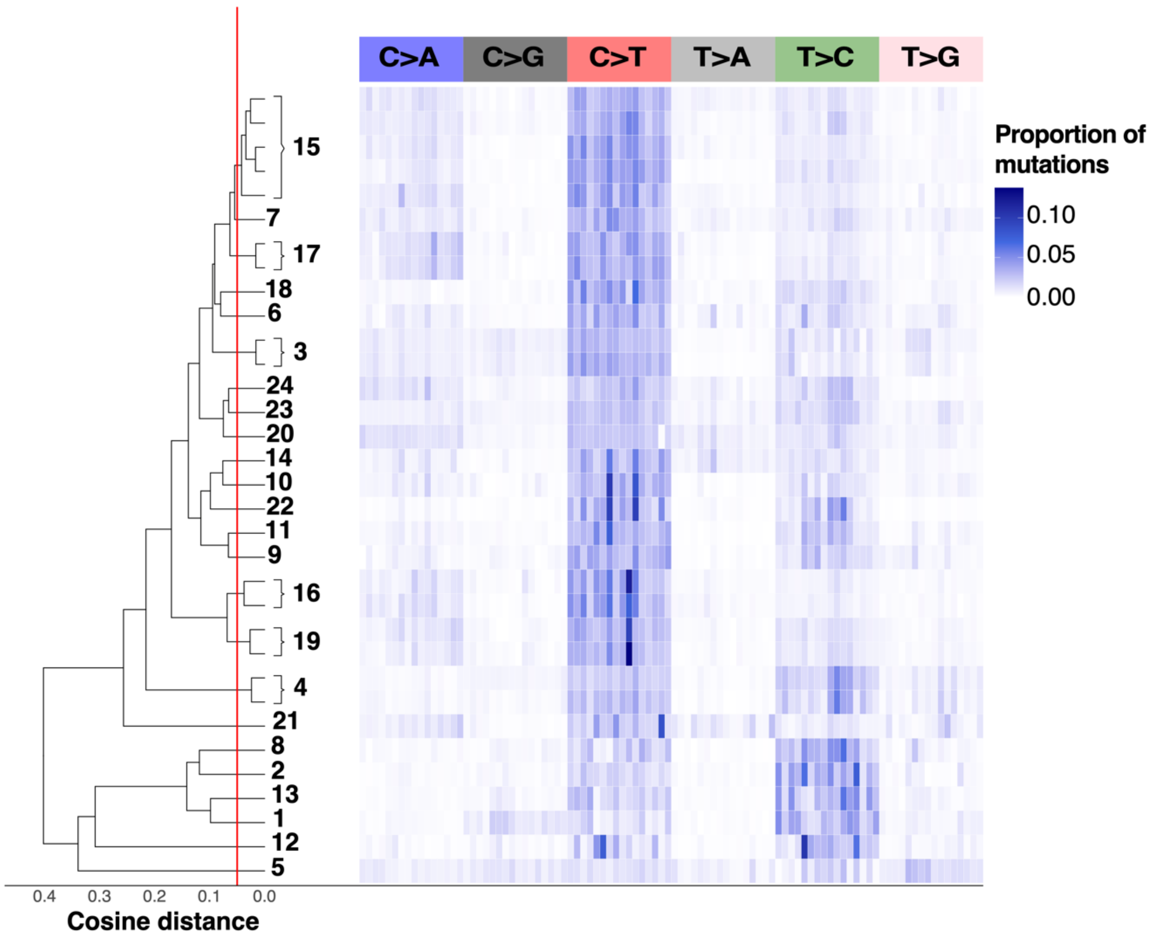

Figure S15. Mutational signatures extracted from decomposition analysis of datasets of species and genus SBS spectra. Hierarchical clustering of the 33 mutational signatures extracted from 13 extraction datasets containing SBS spectra from clades within a species or within a genus. Signatures were combined if they cluster at cosine similarity of 0.95, shown by the red vertical line. Based on this, the 33 extracted mutational signatures were collapsed to 24 final mutational signatures, which are labelled on the corresponding tip branches. The heatmap shows the proportion of contextual mutations within each mutational signature. Source data are provided as a Source Data file.

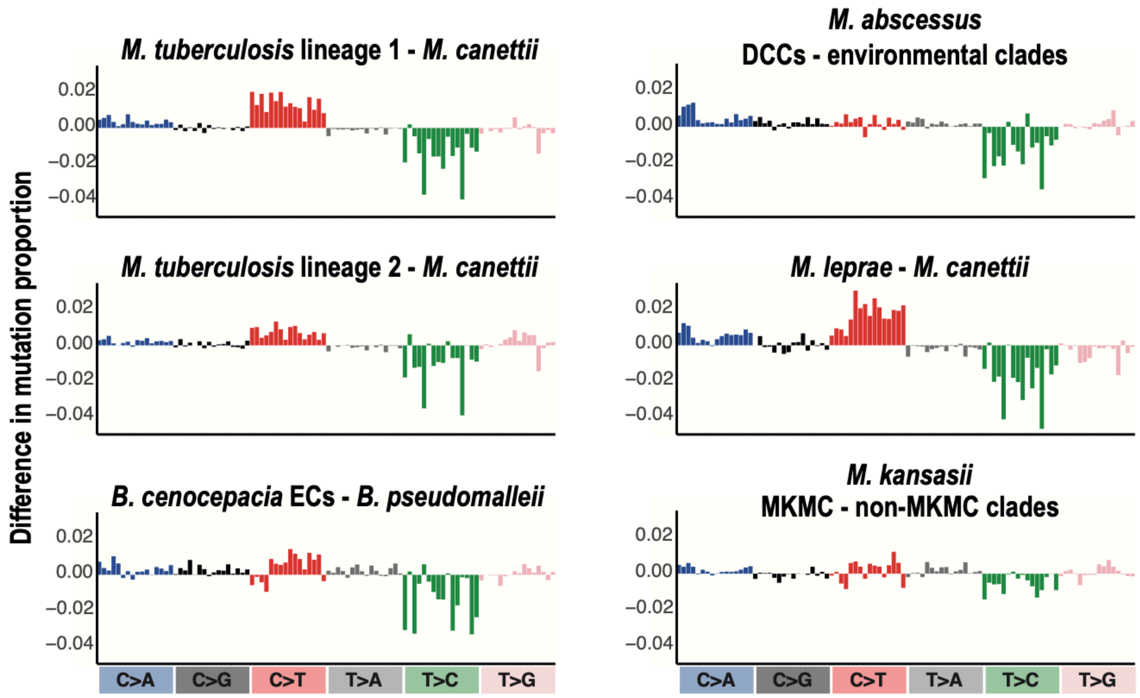

Figure S16. Comparison of mutational spectra between lung and environmental *Mycobacteria* and *Burkholderia*. Subtraction of mutation proportions in SBS spectra between closely related bacterial clades. Each comparison subtracts the SBS spectrum of a known environmental clade from the SBS spectrum of a clade either known to reside within the lung or with an unknown niche. Source data are provided as a Source Data file.

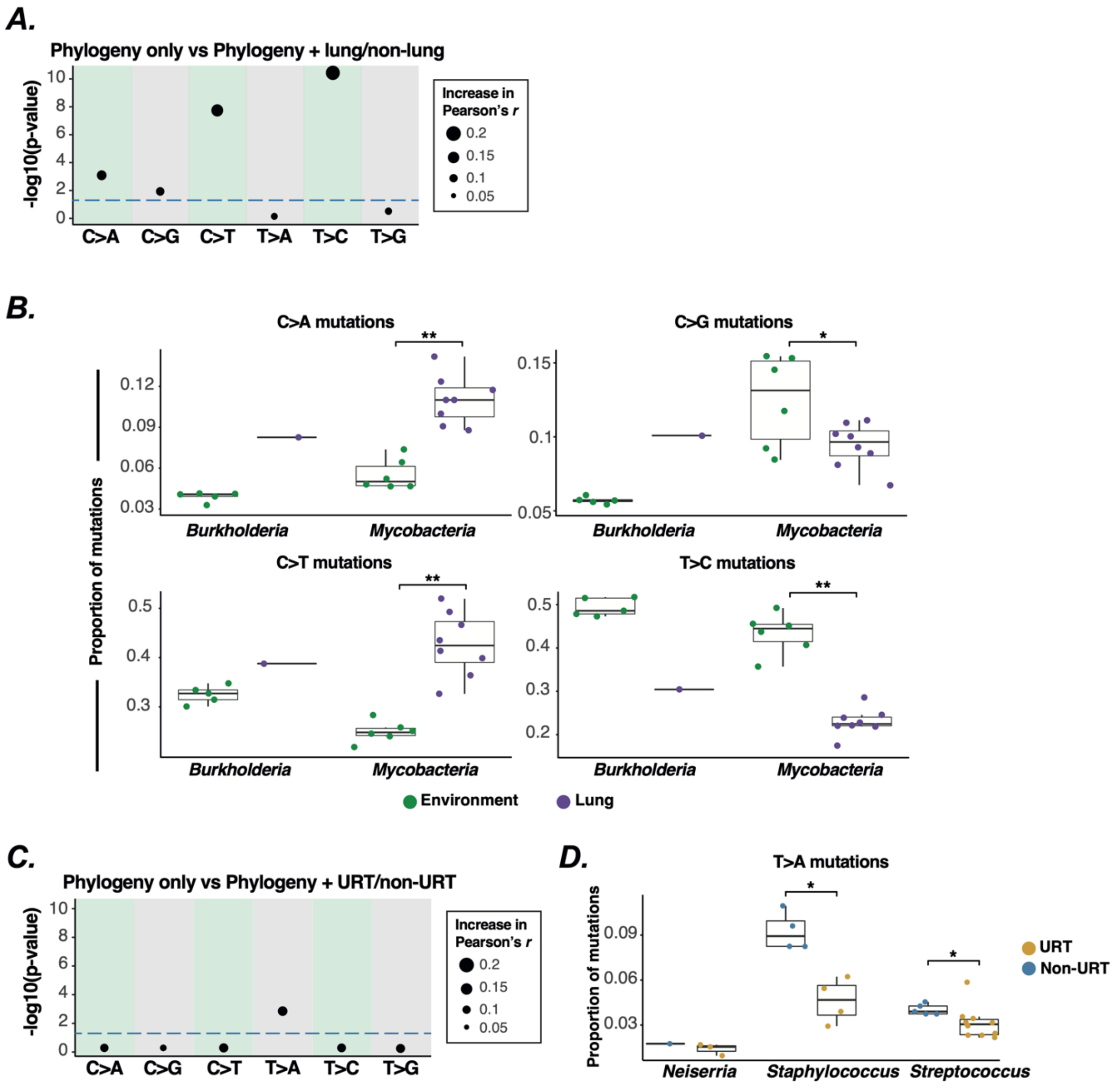

Figure S17. Mutation types associated with pathogen niches. Mutation types with an association with a pathogen niche were identified through linear models, comparing a model with genus only with a model including genus and a binary niche predictor variable. (**A** and **C**) LOD scores showing the significance of the improvement in model fit when including (**A**) lung/environment or (**C**) URT/non-URT as an additional predictor variable compared with genus only. Blue dashed lines correspond to a p-value of 0.05; points above this line exhibit a significant improvement in model fit, suggesting that the respective niche(s) influence that mutation type. Point sizes are proportional to the improvement in R^2 when including the niche. (**B**) Comparison of mutation type proportions between lung and environmental niches in *Mycobacteria* and *Burkholderia*. The proportion of mutations of the respective type is plotted for each spectrum in the respective niche. Significance of differences between *Mycobacteria* niches was calculated through comparing the difference in median proportions in the real data with that in 1000 bootstrap randomisations of niches; * shows $p < 0.05$, ** shows $p < 0.01$. While C>G was detected as significant, there is not a consistent direction of change between *Mycobacteria* and *Burkholderia* so we did not analyse this mutation further. Number of clades analysed (n): *Burkholderia* environment = 5, *Burkholderia* lung = 1, *Mycobacteria* environment = 6, *Mycobacteria* lung = 9. (**D**) Comparison of the proportion of T>A mutations within URT and non-URT clades of *Neisseria*, *Staphylococcus* and *Streptococcus*. As in **Fig. 5A**. Number of clades included: *Neisseria* other = 1, *Neisseria* URT = 3, *Staphylococcus* other = 4, *Staphylococcus* URT = 4, *Streptococcus* other = 5, *Streptococcus* URT = 10. In (**B**) and (**D**), boxplot centre lines show median value; upper and lower bounds show the 25th and 75th quantile, respectively; upper and lower whiskers show the largest and smallest values within 1.5 times the interquartile range above the 75th percentile and below the 25th percentile, respectively. All clade values are shown as points. Source data are provided as a Source Data file.

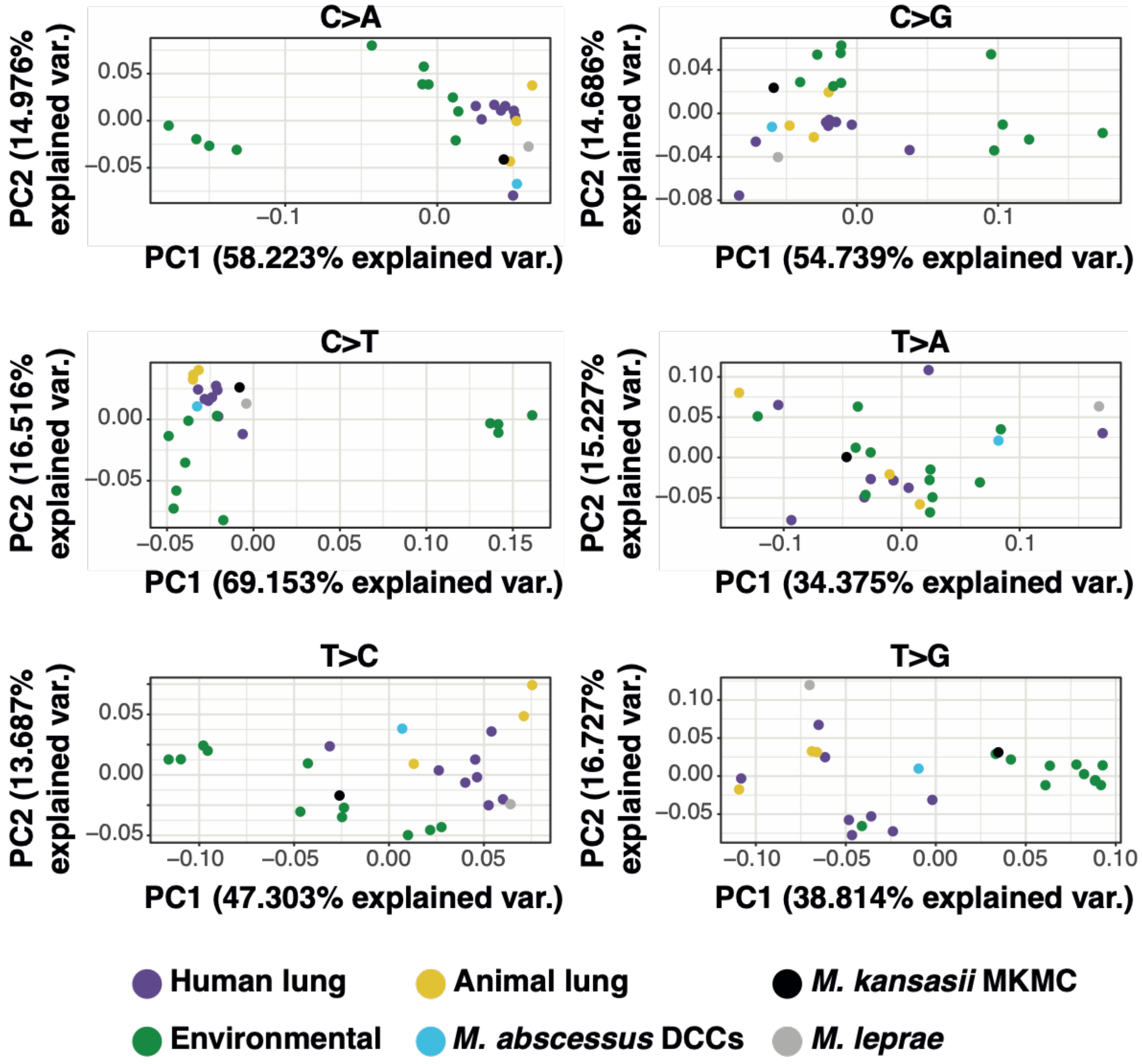

Figure S18. Contextual mutation comparisons between *Mycobacteria* and *Burkholderia*. PCA of contextual mutation proportions within each mutation type across *Mycobacteria* and *Burkholderia* SBS spectra. Source data are provided as a Source Data file.

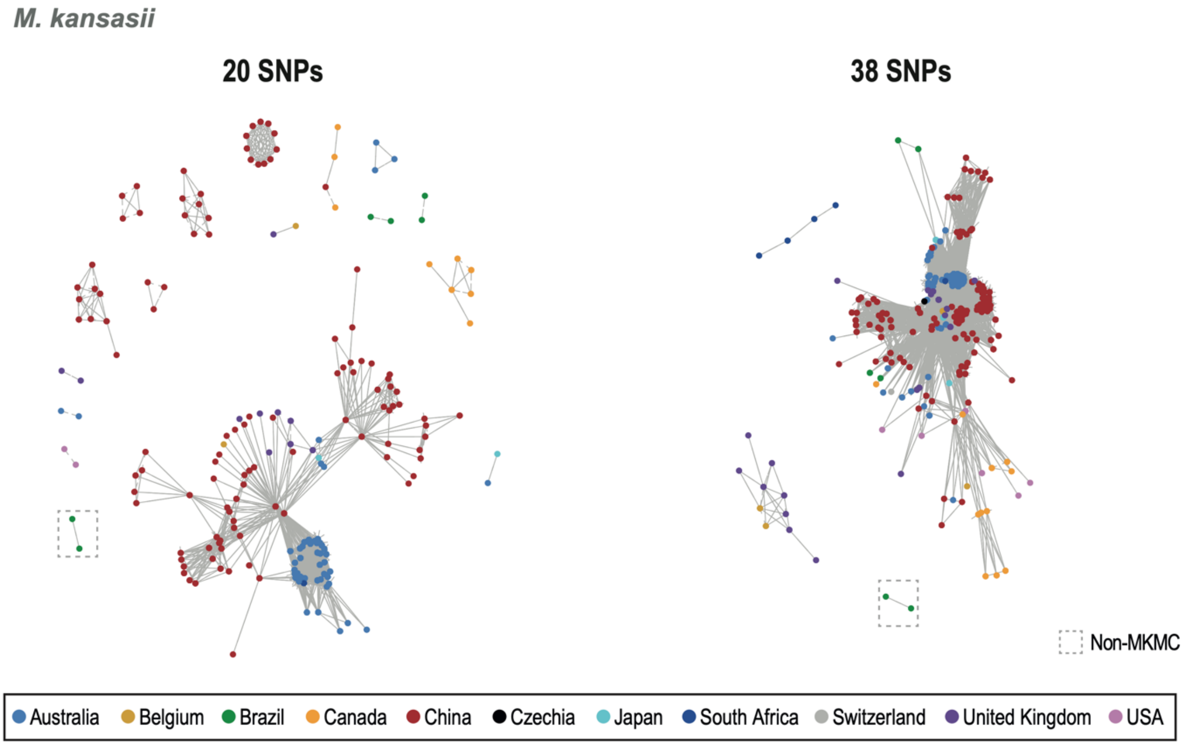

Figure S19. Global transmission networks within *M. kansasii*. Nodes show patients infected with *M. kansasii*, edges show that patient isolates differ by less than or equal to 20 SNPs (left hand panel) or 38 SNPs (right hand panel). Nodes are coloured by the country in which the isolate was collected. The dashed rectangles show a pair of isolates that are not within the MKMC clade. All other isolates cluster within the MKMC.

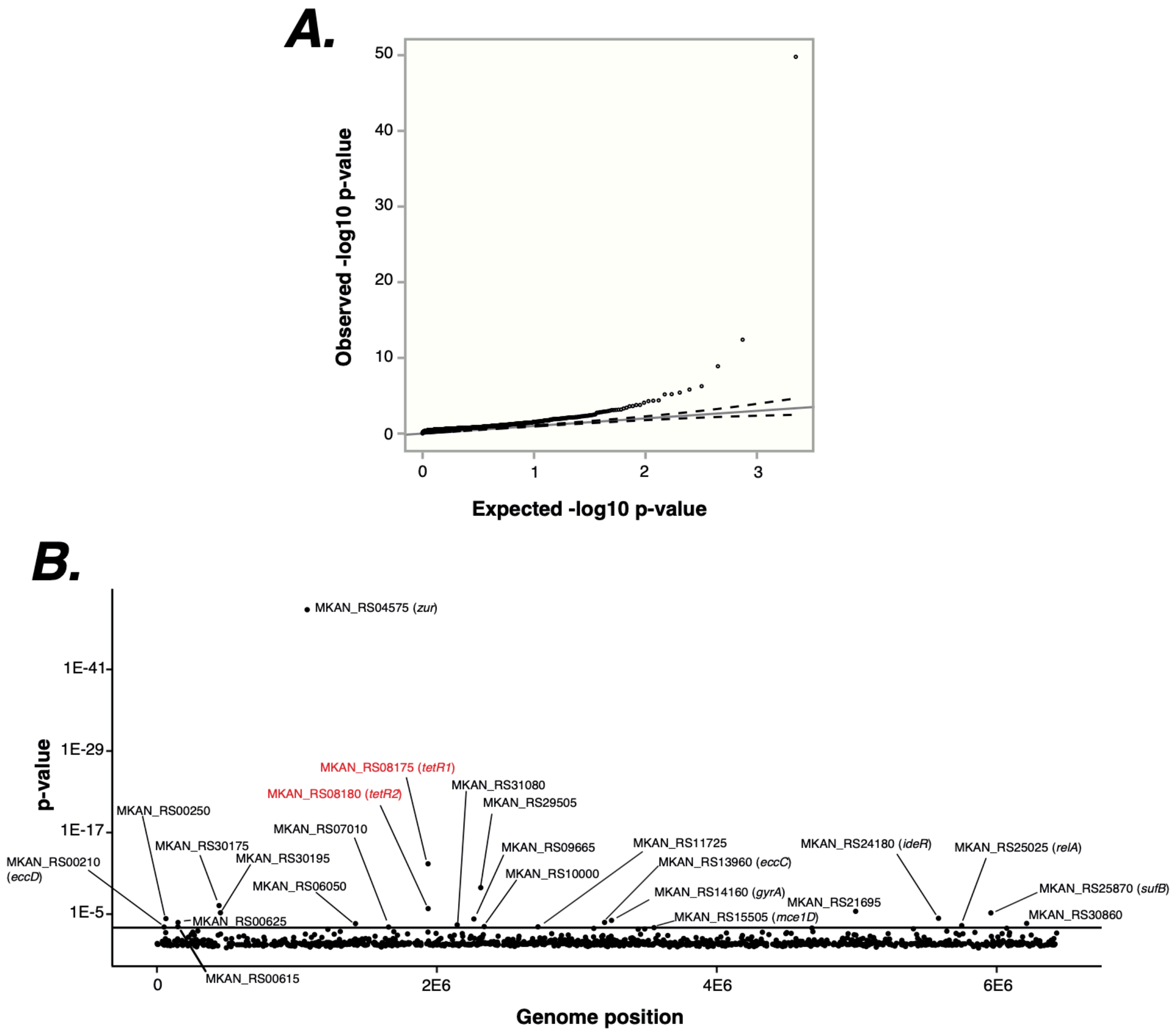

Figure S20. Gene burden test in *M. kansasii*. (**A**) Quantile-quantile plot of gene burden test results. Each point represents a single gene in the genome. The expected -log10 p-value axis shows the expected p-values under a uniform distribution. (**B**) Manhattan plot showing adjusted p-values from the gene burden test across all genes in the *M. kansasii* reference genome (accession NC022663.1). Significance assessed through a Poisson test comparing the expected number of mutations based on gene length and the observed number of mutations in each gene. Genes with significantly more mutations than expected are labelled. The two tetR family genes described in **Fig. S21** are labelled in red. Source data are provided as a Source Data file.

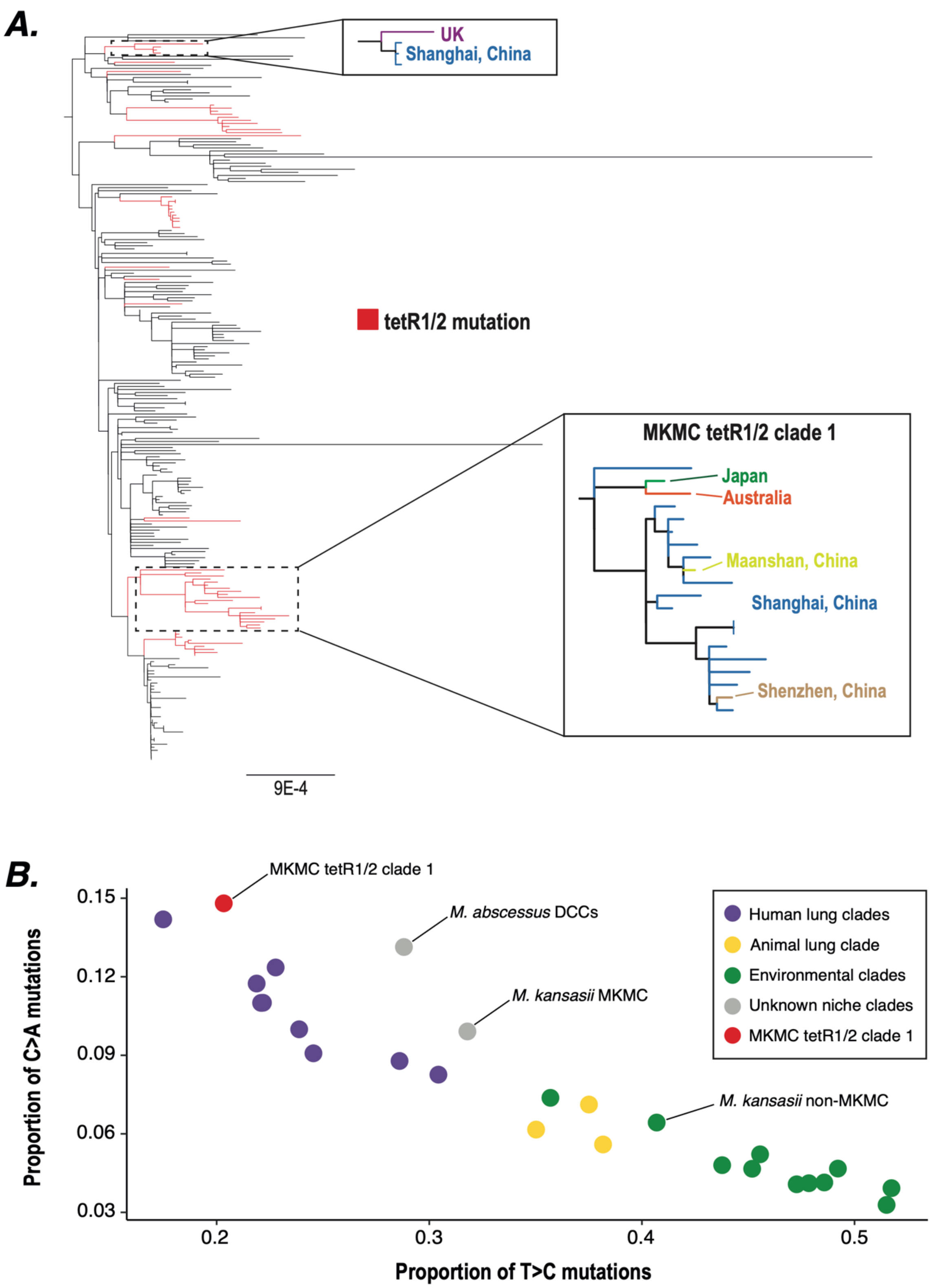

Figure S21. Frequent mutation of *tetR1/2* genes in MKMC. (**A**) Phylogenetic tree of the MKMC clade of *M. kansasii*. Phylogenetic branches coloured red contain a mutation in either the *tetR1* or *tetR2* gene; these genes have mutated on 14 independent branches within the MKMC, including six internal branches, and 26% of MKMC sequences contain a mutation in *tetR1* or *tetR2*. Clades containing a mutation in *tetR1* or *tetR2* that have been isolated in multiple countries are highlighted with tip branches coloured by country of collection. The scale bar shows the expected number of substitution per variable site. (**B**) The proportion of T>C and proportion of C>A mutations is shown in MKMC tetR1/2 clade 1 (highlighted in **panel A**) compared with other clades of *Mycobacteria* and *Burkholderia*. The MKMC tetR1/2 clade 1 contains high C>A mutations and low T>C mutations supporting replication within the lung. Source data are provided as a Source Data file.

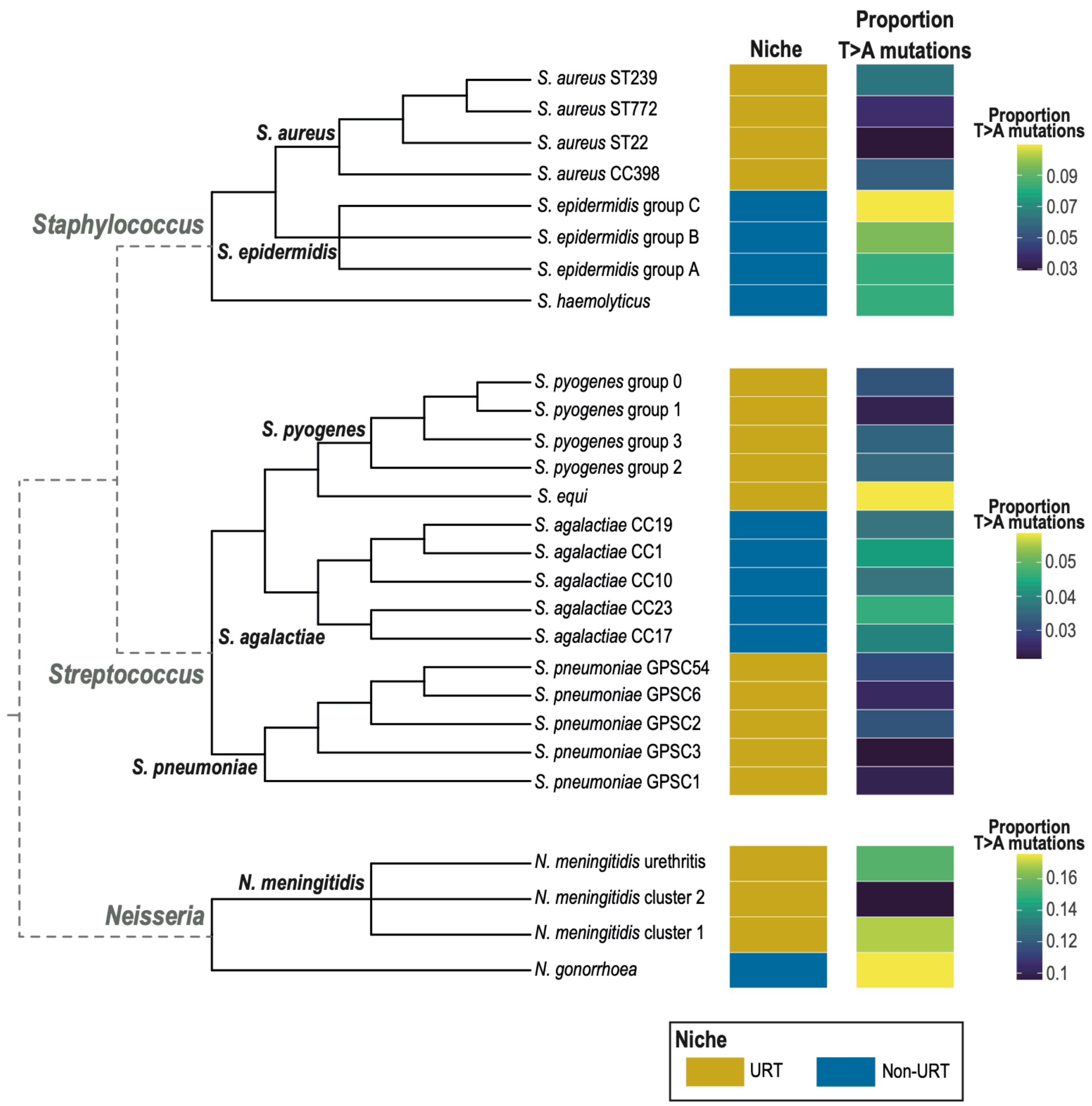

Figure S22. Independent changes in T>A mutations between URT and non-URT clades. Dendrogram shows phylogenetic relationships between *Staphylococcus*, *Streptococcus* and *Neisseria* clades. The left hand heatmap shows niche of each clade; URT shows that the clade is solely or partially found in the URT while non-URT clades are not found in the URT. The right hand heatmap shows the proportion of mutations in the respective SBS spectrum that are T>A. Each of the three independent changes between URT and non-URT is associated with a change in T>A mutations, with URT clades showing significantly reduced levels of T>A mutations. Source data are provided as a Source Data file.

Supplementary References

1. Scoffone, V. C. *et al.* Burkholderia cenocepacia Infections in Cystic Fibrosis Patients: Drug Resistance and Therapeutic Approaches. *Front Microbiol* **8**, 1592 (2017).
2. Lee, A. H.-Y. *et al.* Phenotypic diversity and genotypic flexibility of Burkholderia cenocepacia during long-term chronic infection of cystic fibrosis lungs. *Genome Res.* **27**, 650–662 (2017).
3. Martina, P. *et al.* Hypermutation in Burkholderia cepacia complex is mediated by DNA mismatch repair inactivation and is highly prevalent in cystic fibrosis chronic respiratory infection. *International Journal of Medical Microbiology* **304**, 1182–1191 (2014).
4. Wiersinga, W. J. *et al.* Melioidosis. *Nat Rev Dis Primers* **4**, 1–22 (2018).
5. Chewapreecha, C. *et al.* Genetic variation associated with infection and the environment in the accidental pathogen Burkholderia pseudomallei. *Communications Biology* **2**, 1–11 (2019).
6. Limmathurotsakul, D. *et al.* Predicted global distribution of Burkholderia pseudomallei and burden of melioidosis. *Nat Microbiol* **1**, 1–5 (2016).
7. Kaakoush, N. O., Castaño-Rodríguez, N., Mitchell, H. M. & Man, S. M. Global Epidemiology of Campylobacter Infection. *Clinical Microbiology Reviews* **28**, 687–720 (2015).
8. SHEPPARD, S. K. *et al.* Niche segregation and genetic structure of Campylobacter jejuni populations from wild and agricultural host species. *Mol Ecol* **20**, 3484–3490 (2011).
9. Mourkas, E. *et al.* Agricultural intensification and the evolution of host specialism in the enteric pathogen Campylobacter jejuni. *PNAS* **117**, 11018–11028 (2020).
10. Oh, J. *et al.* Biogeography and individuality shape function in the human skin metagenome. *Nature* **514**, 59–64 (2014).
11. Ahle, C. M., Feidenhansl, C. & Brüggemann, H. Cutibacterium acnes. *Trends in Microbiology* **0**, (2022).
12. Pöntinen, A. K. *et al.* Apparent nosocomial adaptation of Enterococcus faecalis predates the modern hospital era. *Nature Communications* **12**, 1523 (2021).
13. Daniel, D. S., Lee, S. M., Gan, H. M., Dykes, G. A. & Rahman, S. Genetic diversity of Enterococcus faecalis isolated from environmental, animal and clinical sources in Malaysia. *Journal of Infection and Public Health* **10**, 617–623 (2017).

14. Croxen, M. A. *et al.* Recent Advances in Understanding Enteric Pathogenic Escherichia coli. *Clin Microbiol Rev* **26**, 822–880 (2013).

15. Horesh, G. *et al.* A comprehensive and high-quality collection of Escherichia coli genomes and their genes. *Microbial Genomics* **7**, 000499.

16. Holt, K. E. *et al.* Shigella sonnei genome sequencing and phylogenetic analysis indicate recent global dissemination from Europe. *Nat Genet* **44**, 1056–1059 (2012).

17. Connor, T. R. *et al.* Species-wide whole genome sequencing reveals historical global spread and recent local persistence in Shigella flexneri. *eLife* **4**, e07335 (2015).

18. King, P. Haemophilus influenzae and the lung (Haemophilus and the lung). *Clin Transl Med* **1**, 10 (2012).

19. Watts, S. C. & Holt, K. E. hicap: In Silico Serotyping of the Haemophilus influenzae Capsule Locus. *J Clin Microbiol* **57**, e00190-19 (2019).

20. David, S. *et al.* Epidemic of carbapenem-resistant Klebsiella pneumoniae in Europe is driven by nosocomial spread. *Nature Microbiology* **4**, 1919–1929 (2019).

21. Martin, R. M. & Bachman, M. A. Colonization, Infection, and the Accessory Genome of Klebsiella pneumoniae. *Frontiers in Cellular and Infection Microbiology* **8**, (2018).

22. Ashurst, J. V. & Dawson, A. Klebsiella Pneumonia. in *StatPearls* (StatPearls Publishing, 2022).

23. Farida, H. *et al.* Nasopharyngeal Carriage of Klebsiella pneumoniae and Other Gram-Negative Bacilli in Pneumonia-Prone Age Groups in Semarang, Indonesia. *J Clin Microbiol* **51**, 1614–1616 (2013).

24. Raffelsberger, N. *et al.* Gastrointestinal carriage of Klebsiella pneumoniae in a general adult population: a cross-sectional study of risk factors and bacterial genomic diversity. *Gut Microbes* **13**, 1939599.

25. Al-Quadan, T., Price, C. T. & Kwaik, Y. A. Exploitation of evolutionarily conserved amoeba and mammalian processes by Legionella. *Trends Microbiol* **20**, 299–306 (2012).

26. David, S. *et al.* Multiple major disease-associated clones of Legionella pneumophila have emerged recently and independently. *Genome Res.* **26**, 1555–1564 (2016).

27. Ruis, C. *et al.* Dissemination of Mycobacterium abscessus via global transmission networks. *Nat Microbiol* 1–10 (2021) doi:10.1038/s41564-021-00963-3.

28. Bryant, J. M. *et al.* Emergence and spread of a human-transmissible multidrug-resistant nontuberculous mycobacterium. *Science* **354**, 751–757 (2016).

29. Tzou, C. L. *et al.* Association between Mycobacterium avium Complex Pulmonary Disease and Mycobacteria in Home Water and Soil. *Annals ATS* **17**, 57–62 (2020).

30. Uchiya, K. *et al.* Comparative genome analyses of Mycobacterium avium reveal genomic features of its subspecies and strains that cause progression of pulmonary disease. *Scientific Reports* **7**, 39750 (2017).

31. Blouin, Y. *et al.* Progenitor "Mycobacterium canettii" Clone Responsible for Lymph Node Tuberculosis Epidemic, Djibouti - Volume 20, Number 1—January 2014 - Emerging Infectious Diseases journal - CDC. doi:10.3201/eid2001.130652.

32. Supply, P. & Brosch, R. The Biology and Epidemiology of Mycobacterium canettii. in *Strain Variation in the Mycobacterium tuberculosis Complex: Its Role in Biology, Epidemiology and Control* (ed. Gagneux, S.) 27–41 (Springer International Publishing, 2017). doi:10.1007/978-3-319-64371-7_2.

33. Siddam, A. D. *et al.* Characterization of Biofilm Formation by Mycobacterium chimaera on Medical Device Materials. *Front Microbiol* **11**, 586657 (2021).

34. Honda, J. R., Virdi, R. & Chan, E. D. Global Environmental Nontuberculous Mycobacteria and Their Contemporaneous Man-Made and Natural Niches. *Frontiers in Microbiology* **9**, (2018).

35. van Tonder, A. J. *et al. Mycobacterium avium* complex (MAC) genomics and transmission in a London hospital. *Eur Respir J* 2201237 (2022) doi:10.1183/13993003.01237-2022.

36. Luo, T. *et al.* Population genomics provides insights into the evolution and adaptation to humans of the waterborne pathogen Mycobacterium kansasii. *Nat Commun* **12**, 2491 (2021).

37. Ploemacher, T., Faber, W. R., Menke, H., Rutten, V. & Pieters, T. Reservoirs and transmission routes of leprosy; A systematic review. *PLoS Negl Trop Dis* **14**, e0008276 (2020).

38. Benjak, A. *et al.* Phylogenomics and antimicrobial resistance of the leprosy bacillus Mycobacterium leprae. *Nature Communications* **9**, 352 (2018).

39. Gagneux, S. Ecology and evolution of Mycobacterium tuberculosis. *Nat Rev Microbiol* **16**, 202–213 (2018).

40. Smith, R. M. M. *et al.* Mycobacterium bovis Infection, United Kingdom. *Emerg Infect Dis* **10**, 539–541 (2004).

41. Nigsch, A., Glawischnig, W., Bagó, Z. & Greber, N. Mycobacterium caprae Infection of Red Deer in Western Austria–Optimized Use of Pathology Data to Infer Infection Dynamics. *Front. Vet. Sci.* **5**, (2019).

42. Quillin, S. J. & Seifert, H. S. Neisseria gonorrhoeae host-adaptation and pathogenesis. *Nat Rev Microbiol* **16**, 226–240 (2018).

43. Thomas, J. C. *et al.* Phylogenomic analysis reveals persistence of gonococcal strains with reduced-susceptibility to extended-spectrum cephalosporins and mosaic penA-34. *Nat Commun* **12**, 3801 (2021).

44. Caugant, D. A. & Brynildsrud, O. B. Neisseria meningitidis: using genomics to understand diversity, evolution and pathogenesis. *Nat Rev Microbiol* **18**, 84–96 (2020).

45. Bazan, J. A. *et al.* Large Cluster of Neisseria meningitidis Urethritis in Columbus, Ohio, 2015. *Clin Infect Dis* **65**, 92–99 (2017).

46. Streeter, K. & Katouli, M. Pseudomonas aeruginosa: A review of their Pathogenesis and Prevalence in Clinical Settings and the Environment. *Infection Epidemiology and Microbiology* **2**, 25–32 (2016).

47. Crone, S. *et al.* The environmental occurrence of Pseudomonas aeruginosa. *APMIS* **128**, 220–231 (2020).

48. Okoro, C. K. *et al.* Intracontinental spread of human invasive Salmonella Typhimurium pathovariants in sub-Saharan Africa. *Nature Genetics* **44**, 1215–1221 (2012).

49. Achtman, M. *et al.* Multilocus Sequence Typing as a Replacement for Serotyping in Salmonella enterica. *PLOS Pathogens* **8**, e1002776 (2012).

50. Peacock, S. J., de Silva, I. & Lowy, F. D. What determines nasal carriage of Staphylococcus aureus? *Trends in Microbiology* **9**, 605–610 (2001).

51. Wertheim, H. F. *et al.* The role of nasal carriage in Staphylococcus aureus infections. *The Lancet Infectious Diseases* **5**, 751–762 (2005).

52. Turner, N. A. *et al.* Methicillin-resistant Staphylococcus aureus: an overview of basic and clinical research. *Nat Rev Microbiol* **17**, 203–218 (2019).
53. Holden, M. T. G. *et al.* A genomic portrait of the emergence, evolution, and global spread of a methicillin-resistant Staphylococcus aureus pandemic. *Genome Res.* **23**, 653–664 (2013).
54. Harris, S. R. *et al.* Evolution of MRSA During Hospital Transmission and Intercontinental Spread. *Science* **327**, 469–474 (2010).
55. Steinig, E. J. *et al.* Evolution and Global Transmission of a Multidrug-Resistant, Community-Associated Methicillin-Resistant Staphylococcus aureus Lineage from the Indian Subcontinent. *mBio* **10**, (2019).
56. Matuszewska, M. *et al.* Stable antibiotic resistance and rapid human adaptation in livestock-associated MRSA. *eLife* **11**, e74819 (2022).
57. Méric, G. *et al.* Disease-associated genotypes of the commensal skin bacterium Staphylococcus epidermidis. *Nature Communications* **9**, 5034 (2018).
58. Miragaia, M., Thomas, J. C., Couto, I., Enright, M. C. & de Lencastre, H. Inferring a Population Structure for Staphylococcus epidermidis from Multilocus Sequence Typing Data. *Journal of Bacteriology* **189**, 2540–2552 (2007).
59. Garza-González, E., Morfín-Otero, R., Llaca-Díaz, J. M. & Rodriguez-Noriega, E. Staphylococcal cassette chromosome mec (SCCmec) in methicillin-resistant coagulase-negative staphylococci. A review and the experience in a tertiary-care setting. *Epidemiology & Infection* **138**, 645–654 (2010).
60. Takeuchi, F. *et al.* Whole-Genome Sequencing of Staphylococcus haemolyticus Uncovers the Extreme Plasticity of Its Genome and the Evolution of Human-Colonizing Staphylococcal Species. *Journal of Bacteriology* **187**, 7292 (2005).
61. Cavanagh, J. P. *et al.* Whole-genome sequencing reveals clonal expansion of multiresistant Staphylococcus haemolyticus in European hospitals. *Journal of Antimicrobial Chemotherapy* **69**, 2920–2927 (2014).
62. Schuchat, A. Group B streptococcus. *The Lancet* **353**, 51–56 (1999).

63. Landwehr-Kenzel, S. & Henneke, P. Interaction of Streptococcus agalactiae and Cellular Innate Immunity in Colonization and Disease. *Front Immunol* **5**, 519 (2014).

64. Jamrozy, D. *et al.* Increasing incidence of group B streptococcus neonatal infections in the Netherlands is associated with clonal expansion of CC17 and CC23. *Scientific Reports* **10**, 9539 (2020).

65. Da Cunha, V. *et al.* Streptococcus agalactiae clones infecting humans were selected and fixed through the extensive use of tetracycline. *Nat Commun* **5**, 4544 (2014).

66. Boyle, A. G. *et al.* Streptococcus equi Infections in Horses: Guidelines for Treatment, Control, and Prevention of Strangles—Revised Consensus Statement. *J Vet Intern Med* **32**, 633–647 (2018).

67. Mitchell, C. *et al.* Globetrotting strangles: the unbridled national and international transmission of Streptococcus equi between horses. *Microbial Genomics* **7**, 000528.

68. Weiser, J. N., Ferreira, D. M. & Paton, J. C. Streptococcus pneumoniae: transmission, colonization and invasion. *Nat Rev Microbiol* **16**, 355–367 (2018).

69. Gladstone, R. A. *et al.* International genomic definition of pneumococcal lineages, to contextualise disease, antibiotic resistance and vaccine impact. *EBioMedicine* **43**, 338–346 (2019).

70. Bessen, D. E. Population Biology of the Human Restricted Pathogen, Streptococcus pyogenes. *Infect Genet Evol* **9**, 581–593 (2009).

71. Davies, M. R. *et al.* Atlas of group A streptococcal vaccine candidates compiled using large-scale comparative genomics. *Nat Genet* **51**, 1035–1043 (2019).

72. Stenseth, N. C. *et al.* Plague: Past, Present, and Future. *PLoS Med* **5**, e3 (2008).

73. Dubyanskiy, V. M. & Yeszhanov, A. B. Ecology of Yersinia pestis and the Epidemiology of Plague. in *Yersinia pestis: Retrospective and Perspective* (eds. Yang, R. & Anisimov, A.) 101–170 (Springer Netherlands, 2016). doi:10.1007/978-94-024-0890-4_5.

74. Hinnebusch, B. J., Jarrett, C. O. & Bland, D. M. “Fleaing” the Plague: Adaptations of Yersinia pestis to Its Insect Vector That Lead to Transmission. *Annual Review of Microbiology* **71**, 215–232 (2017).

75. Cui, Y. *et al.* Historical variations in mutation rate in an epidemic pathogen, Yersinia pestis. *Proceedings of the National Academy of Sciences* **110**, 577–582 (2013).

76. Bryant, J. M. *et al.* Whole-genome sequencing to identify transmission of Mycobacterium abscessus between patients with cystic fibrosis: a retrospective cohort study. *The Lancet* **381**, 1551–1560 (2013).
77. Bryant, J. M. *et al.* Stepwise pathogenic evolution of Mycobacterium abscessus. *Science* **372**, (2021).
