# Supplementary material for: Mutational spectra are associated with bacterial niche
Source: Nat Commun. 2023 Nov 4;14:7091. doi: 10.1038/s41467-023-42916-w (PMC10625568; doi:10.1038/s41467-023-42916-w)
Supplement: Supplementary file 3 — Description of Additional Supplementary Files [file 41467_2023_42916_MOESM3_ESM.pdf]

Description of Additional Supplementary Files:

Supplementary Data 1: Datasets used for reconstruction of SBS and DBS mutational spectra.

Supplementary Data 2: SBS spectra calculated from the 84 bacterial clades.

Supplementary Data 3: Datasets used for NMF signature extraction.

Supplementary Data 4: Bacteria SBS signatures and the datasets they were extracted from.

Supplementary Data 5: Association of SBS mutations with pathogen niche.

Supplementary Data 6: Accession numbers of samples used in mutational spectrum datasets.

Supplementary Data 7: Modelling of DNA polymerase III subunits in Pseudomonas aeruginosa and Burkholderia cenocepacia.

Supplementary Data 8: Summary of comparisons of linear models incorporating genus with linear models including genus and niche.
