# Supplementary material for: Mutational spectra are associated with bacterial niche
Source: Nat Commun. 2023 Nov 4;14:7091. doi: 10.1038/s41467-023-42916-w (PMC10625568; doi:10.1038/s41467-023-42916-w)
Supplement: Supplementary file 12 — Reporting Summary [file 41467_2023_42916_MOESM12_ESM.pdf]

nature portfolio

Corresponding author(s): Julian Parkhill, R. Andres Floto

Last updated by author(s): Oct 12, 2023

Reporting Summary

Nature Portfolio wishes to improve the reproducibility of the work that we publish. This form provides structure for consistency and transparency in reporting. For further information on Nature Portfolio policies, see our Editorial Policies and the Editorial Policy Checklist.

Statistics

For all statistical analyses, confirm that the following items are present in the figure legend, table legend, main text, or Methods section.

n/a	Confirmed	
☐	☒	The exact sample size (*n*) for each experimental group/condition, given as a discrete number and unit of measurement
☐	☒	A statement on whether measurements were taken from distinct samples or whether the same sample was measured repeatedly
☐	☒	The statistical test(s) used AND whether they are one- or two-sided<br>*Only common tests should be described solely by name; describe more complex techniques in the Methods section.*
☒	☐	A description of all covariates tested
☐	☒	A description of any assumptions or corrections, such as tests of normality and adjustment for multiple comparisons
☐	☒	A full description of the statistical parameters including central tendency (e.g. means) or other basic estimates (e.g. regression coefficient) AND variation (e.g. standard deviation) or associated estimates of uncertainty (e.g. confidence intervals)
☒	☐	For null hypothesis testing, the test statistic (e.g. *F*, *t*, *r*) with confidence intervals, effect sizes, degrees of freedom and *P* value noted<br>*Give P values as exact values whenever suitable.*
☒	☐	For Bayesian analysis, information on the choice of priors and Markov chain Monte Carlo settings
☒	☐	For hierarchical and complex designs, identification of the appropriate level for tests and full reporting of outcomes
☐	☒	Estimates of effect sizes (e.g. Cohen's *d*, Pearson's *r*), indicating how they were calculated

Our web collection on statistics for biologists contains articles on many of the points above.

Software and code

Policy information about availability of computer code

Data collection	No software was used in the collection of the data
Data analysis	Genome assemblies were shredded to FASTQ files using Fastaq v3.17.0. Sequencing reads were mapped against a reference genome using the multiple_mappings_to_bam pipeline v1.6. Recombination was removed from sequence alignments using Gubbins v2.4.1. Maximum likelihood phylogenetic trees were reconstructed using RAxML v8.2.12. Mutational spectra were reconstructed using MutTui v1.1.10 incorporating ancestral sequence reconstruction through treetime v0.8.1. Overall SBS spectra were compared using the R package umap v0.2.7.0. DNA repair signatures were fit to Campylobacter-elevated mutations using the R package signature.tools.lib v2.1.2. AlphaFold v2.0 was used to build sequence models. Mutational signatures were extracted using SigProfilerExtractor v1.1.0. The MutTui pipeline is available at https://github.com/chrisruis/MutTui. Additional custom scripts are available at https://doi.org/10.5281/zenodo.8435731.

For manuscripts utilizing custom algorithms or software that are central to the research but not yet described in published literature, software must be made available to editors and reviewers. We strongly encourage code deposition in a community repository (e.g. GitHub). See the Nature Portfolio guidelines for submitting code & software for further information.

Data

Policy information about availability of data

All manuscripts must include a data availability statement. This statement should provide the following information, where applicable:

- Accession codes, unique identifiers, or web links for publicly available datasets
- A description of any restrictions on data availability
- For clinical datasets or third party data, please ensure that the statement adheres to our policy

The datasets generated and analysed during this study have been deposited at https://doi.org/10.5281/zenodo.8435731. All source data, including sequence alignments, phylogenetic trees, reference sequences and mutational spectra are available at https://doi.org/10.5281/zenodo.8435731. Accession numbers of all sequences used in this study are provided in Supplementary Data 6. Human mutational signatures were obtained from the COSMIC database (https://cancer.sanger.ac.uk/signatures/). Source data are provided with this paper.

Research involving human participants, their data, or biological material

Policy information about studies with human participants or human data. See also policy information about sex, gender (identity/presentation), and sexual orientation and race, ethnicity and racism.

Reporting on sex and gender	N/A
Reporting on race, ethnicity, or other socially relevant groupings	N/A
Population characteristics	N/A
Recruitment	N/A
Ethics oversight	N/A

Note that full information on the approval of the study protocol must also be provided in the manuscript.

Field-specific reporting

Please select the one below that is the best fit for your research. If you are not sure, read the appropriate sections before making your selection.

☐ Life sciences ☐ Behavioural & social sciences ☒ Ecological, evolutionary & environmental sciences

For a reference copy of the document with all sections, see nature.com/documents/nr-reporting-summary-flat.pdf

Ecological, evolutionary & environmental sciences study design

All studies must disclose on these points even when the disclosure is negative.

Study description	Mutational spectrum calculation across bacterial clades. Comparison and clustering of mutational spectra. Extraction of DNA repair gene mutational signatures from hypermutator lineages. Decomposition of mutational spectra into signatures.
Research sample	Mutational spectra were reconstructed using genome sequencing datasets from 84 bacterial clades across 31 bacterial species, chosen to represent a broad genetic and ecological diversity of bacteria that have available genome sequencing datasets. This dataset represents the mutational spectra of bacteria from a broad range of phylogenetic diversity and ecological niches. DNA repair gene signatures were calculated from 50 naturally-occurring bacterial hypermutator lineages; this includes all of the identified hypermutator lineages across the dataset. Datasets were previously published and we obtained them either from NCBI GenBank as assemblies or from authors of previous studies.
Sampling strategy	We included all sequences from previously published datasets across 84 bacterial clades
Data collection	Sequencing datasets were obtained either from public databases as FASTQ files or genome assemblies, or from previous study Authors as genome sequence alignments or post-recombination removal variable sites alignments
Timing and spatial scale	We included all sequences from previously published datasets across 84 bacterial clades. These sequences were collected from all continents and over several decades
Data exclusions	No data were excluded from the analyses
Reproducibility	Mutational spectra were calculated on six individual subclades of Neisseria gonorrhoeae and compared with the complete clade; all spectra were highly similar
Randomization	Sequences were divided into clades based on phylogenetic relationships

Blinding	All samples were coded by unique lab identification and therefore all associated metadata are anonymised

Did the study involve field work? ☐ Yes ☒ No

Reporting for specific materials, systems and methods

We require information from authors about some types of materials, experimental systems and methods used in many studies. Here, indicate whether each material, system or method listed is relevant to your study. If you are not sure if a list item applies to your research, read the appropriate section before selecting a response.

Materials & experimental systems

n/a	Involved in the study
☒	☐ Antibodies
☒	☐ Eukaryotic cell lines
☒	☐ Palaeontology and archaeology
☒	☐ Animals and other organisms
☒	☐ Clinical data
☒	☐ Dual use research of concern
☒	☐ Plants

Methods

n/a	Involved in the study
☒	☐ ChIP-seq
☒	☐ Flow cytometry
☒	☐ MRI-based neuroimaging

Plants

Seed stocks	*Report on the source of all seed stocks or other plant material used. If applicable, state the seed stock centre and catalogue number. If plant specimens were collected from the field, describe the collection location, date and sampling procedures.*
Novel plant genotypes	*Describe the methods by which all novel plant genotypes were produced. This includes those generated by transgenic approaches, gene editing, chemical/radiation-based mutagenesis and hybridization. For transgenic lines, describe the transformation method, the number of independent lines analyzed and the generation upon which experiments were performed. For gene-edited lines, describe the editor used, the endogenous sequence targeted for editing, the targeting guide RNA sequence (if applicable) and how the editor was applied.*
Authentication	*Describe any authentication procedures for each seed stock used or novel genotype generated. Describe any experiments used to assess the effect of a mutation and, where applicable, how potential secondary effects (e.g. second site T-DNA insertions, mosiacism, off-target gene editing) were examined.*
